# Wafer‐Scale 2D High‐Entropy Transition Metal Dichalcogenide Thin‐Film Catalysts for Efficient and Durable Photoelectrochemical Hydrogen Production

**DOI:** 10.1002/adma.73236

**Published:** 2026-05-06

**Authors:** Sang Eon Jun, Jin Ho Seo, Jaehyun Kim, Hyungsoo Lee, Seongbeen Kim, Woo Seok Cheon, Sabina Kim, In Hye Kwak, Byeong‐Gwan Cho, Ki Chang Kwon, Chul‐Ho Lee, Jungwon Park, Jooho Moon, Jennifer A. Dionne, Ho Won Jang

**Affiliations:** ^1^ Department of Materials Science and Engineering Research Institute of Advanced Materials Seoul National University Seoul South Korea; ^2^ Department of Materials Science and Engineering Stanford University Stanford California USA; ^3^ Department of Materials Science and Engineering Yonsei University Seoul Republic of Korea; ^4^ Department of Chemical and Biomolecular Engineering Korea Advanced Institute of Science and Technology (KAIST) Daejeon Republic of Korea; ^5^ Korea Basic Science Institute Daejeon Republic of Korea; ^6^ Division of Chemical and Material Metrology Korea Research Institute of Standards and Science (KRISS) Daejeon Republic of Korea; ^7^ Department of Applied Measurement Science University of Science and Technology (UST) Daejeon Republic of Korea; ^8^ Department of Electrical and Computer Engineering Seoul National University Seoul Republic of Korea; ^9^ Inter‐university Semiconductor Research Center Seoul National University Seoul Republic of Korea; ^10^ School of Chemical and Biological Engineering Institute of Chemical Processes Seoul National University Seoul Republic of Korea; ^11^ Center for Nanoparticle Research Institute for Basic Science (IBS) Seoul Republic of Korea; ^12^ Institute of Engineering Research College of Engineering Seoul National University Seoul Republic of Korea; ^13^ Advanced Institutes of Convergence Technology Seoul National University Gyeonggi‐do Republic of Korea; ^14^ Department of Radiology Stanford University Stanford California USA

**Keywords:** 2D materials, high entropy materials, hydrogen evolution reaction, photoelectrochemical water splitting, transition metal dichalcogenides

## Abstract

Photoelectrochemical (PEC) performance of conventional 2D transition metal dichalcogenides (TMDs) in hydrogen evolution reaction (HER) is constrained by the limited selection of metal cations, predominantly MoS_2_, whose inert basal planes and unstable 1T phases hinder PEC efficiency. High‐entropy TMDs, in which local lattice distortion and charge redistribution occur within a van der Waals layered structure, are expected to overcome these intrinsic limitations by improving catalytic activity, photocarrier dynamics, and phase stability. Here, we demonstrate a wafer‐scale 2D high‐entropy (MoWTaNbRu)S_2_ thin‐film catalyst with distorted 1T phase on *p*‐Si photocathode for PEC‐HER. The high‐entropy effect induces substantial electronic redistribution, enhancing the contribution of *d*‐orbitals near the Fermi level and optimizing hydrogen adsorption energetics. PEC kinetic analyses, including intensity‐modulated photocurrent spectroscopy, demonstrate that (MoWTaNbRu)S_2_ markedly suppresses the recombination of photogenerated charge carriers, enabling more efficient charge extraction and accelerated interfacial reaction kinetics. Furthermore, the high‐entropy‐driven stabilization of the metastable 1T phase ensures excellent durability of the photocathode. As a result, the (MoWTaNbRu)S_2_/TiO_2_/*p*‐Si photocathode shows a remarkable photocurrent density and stability for over 100 h, outperforming single‐metal TMDs. This study demonstrates how configurational entropy enhances catalytic activity, photocarrier transport, and phase stability of TMDs, establishing a general design principle for next‐generation PEC catalysts.

## Graphical abstract

A wafer‐scale 2D high‐entropy (MoWTaNbRu)S_2_ thin‐film catalyst with a distorted 1T phase integrated onto *p*‐Si photocathode is designed for highly efficient and durable photoelectrochemical hydrogen evolution reaction. The high‐entropy effect in layered transition metal dichalcogenides optimizes hydrogen adsorption energetics, accelerates photogenerated carrier dynamics, and stabilizes the metastable 1T phase, outperforming single‐metal TMDs.

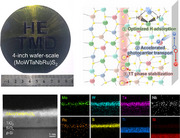

## 1 Introduction

Hydrogen is recognized as a sustainable energy carrier with the potential to decarbonize energy‐intensive industries, yet its widespread deployment depends on the advancement of efficient and durable production technologies [1, 2]. Photoelectrochemical (PEC) water splitting is a promising approach for converting intermittent solar energy into hydrogen fuel [3, 4, 5, 6]. Si has attracted significant attention due to its high absorption coefficient, superior carrier mobility, natural abundance, and well‐established fabrication infrastructure [7, 8, 9]. Despite recent advances, however, integrating Si photoelectrodes into practical monolithic PEC cells remains challenging, primarily due to challenges in simultaneously achieving high quantum efficiency, long‐term operational stability, and scalability [10, 11]. In this regard, the development of highly active, durable, charge‐mediating, and low‐cost thin‐film catalysts that are compatible with scalable coating techniques is of critical importance.

2D transition metal dichalcogenides (2D TMDs) have received significant attention as catalysts for the PEC hydrogen evolution reaction (HER), providing active sites for hydrogen adsorption/desorption and enabling efficient photoinduced electron transport [12, 13, 14]. However, the practical employment of conventional TMDs is constrained by the narrow range of metal cations suitable for HER catalysis, primarily MoS_2_, whose basal planes are intrinsically inert, and metastable active phases suffer from instability [15]. Additionally, low hydrophilicity [16], parasitic light absorption [17], sluggish out‐of‐plane charge transport [18, 19], and the high cost of noble metal‐based TMDs such as PtSe_2_ limit the practical application of single‐element TMDs in PEC‐HER [20].

High‐entropy materials, characterized by the incorporation of multiple principal elements randomly distributed within a single‐phase crystal lattice, have emerged as an ideal platform for electrocatalysis [21, 22, 23]. Their large configurational entropy can induce substantial lattice distortions and promote charge redistribution, enhancing electrocatalytic activity and durability [24, 25]. Extending this concept to 2D TMDs, where local distortions and charge redistribution occur within a van der Waals layered framework, is expected to create new opportunities in photoelectrocatalysis by improving catalytic activity, photocarrier dynamics, and stability [26].

Here, we demonstrate a wafer‐scale 2D high‐entropy (MoWTaNbRu)S_2_ thin‐film catalyst with distorted 1T phase integrated onto *p*‐Si photocathode for highly efficient PEC hydrogen production. Large‐scale, moderate‐temperature, and substrate‐compatible fabrication of HETMD thin films is enabled by thermolysis‐driven synthesis, overcoming the limitations of conventional CVD methods. We selected Mo, W, Ta, Nb, and Ru as constituent metals: Mo and W serve as benchmark TMD hosts with well‐characterized catalytic properties, Ta and Nb introduce metallic conductivity, and Ru provides intrinsic mass activity for HER [27, 28]. Entropy‐driven electronic redistribution enhances the dominance of *d*‐orbitals at the Fermi level, optimizing hydrogen adsorption energetics. Photocarrier dynamic analyses, including intensity‐modulated photocurrent spectroscopy (IMPS), reveal that high‐entropy (MoWTaNbRu)S_2_ effectively suppresses the photo‐induced charge carrier recombination and accelerates the charge transport at the surface. As a result, the (MoWTaNbRu)S_2_/TiO_2_/*p*‐Si photocathode exhibits a remarkable photocurrent density of −21.1 mA cm^−2^ at 0 V versus RHE, outperforming single‐metal TMDs. We further demonstrate that the high‐entropy effect stabilizes the 1T phase, enabling stable operation of the photocathode for over 100 h. This study highlights entropy‐driven enhancement of intrinsic catalytic activity, photocarrier transport, and metastable phase stability in TMDs, broadening their applicability for next‐generation PEC catalysts and devices.

## 2 Results

### 2.1 Wafer‐Scale Thermolysis‐Driven Synthesis of High‐Entropy (MoWTaNbRu)S_2_ Thin Film

**FIGURE 1:**
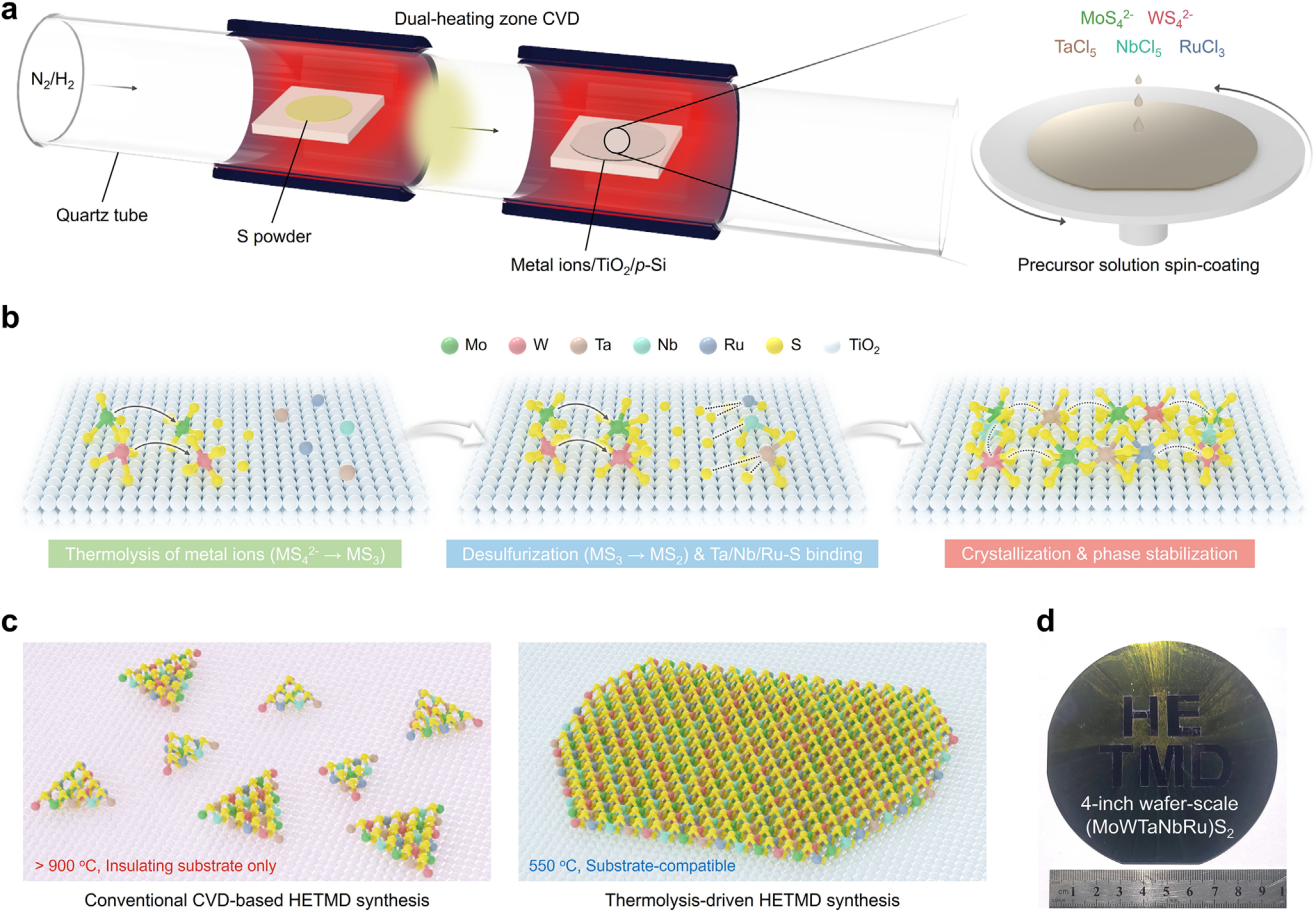
Wafer‐scale thermolysis‐driven synthesis of HETMD thin films. (a) Schematic illustration of the spin‐coating of a mixed precursor solution followed by annealing under N_2_/H_2_ atmosphere with sulfur flow. (b) Growth mechanism for the thermolysis‐driven HETMD synthesis. (c) Comparison between conventional CVD‐based and thermolysis‐driven HETMD synthesis. (d) 4‐inch wafer‐scale high‐entropy (MoWTaNbRu)S_2_ thin film synthesized via the thermolysis‐driven method.

The synthetic process begins with spin‐coating a homogeneous mixed precursor solution containing (NH_4_)_2_MoS_4_, (NH_4_)_2_WS_4_, TaCl_5_, NbCl_5_, and RuCl_3_·xH_2_O dissolved in ethylene glycol onto the substrates (Figure 1a). When the solvent evaporates, the metal ions become randomly distributed within a confined thin layer. Subsequent thermal treatment under sulfur and hydrogen atmosphere drives sequential thermolysis, sulfurization, and crystallization. As illustrated in Figure 1b, during the initial heating stage (100–280°C), the (NH_4_)_2_MoS_4_ and (NH_4_)_2_WS_4_ undergo thermolysis to form MoS_3_ and WS_3_ intermediates [29]. Further annealing up to 470°C induces desulfurization of these intermediates into MoS_2_ and WS_2_, accompanied by the release of S and H_2_S gases. These sulfur species and additional sulfur flow from another heating zone serve as reactive sources that promote sulfurization of adjacent Ta, Nb, and Ru ions to their corresponding sulfides. Finally, annealing at 550°C leads to the crystallization and phase stabilization of a homogeneous high‐entropy (MoWTaNbRu)S_2_. Recently reported CVD methods for HETMDs remain confined to micrometer‐scale domains, limited film uniformity, ultrahigh growth temperatures (>900°C), and narrow substrate compatibility, typically restricted to insulating oxide substrates (Figure 1c; Table S1) [30, 31, 32, 33]. However, our strategy enables the wafer‐scale synthesis of HETMD thin films at relatively low temperatures and on a wide range of substrates. As shown in Figure 1d and Figure S1, we demonstrate the successful synthesis of 4‐inch wafer‐scale high‐entropy (MoWTaNbRu)S_2_ thin film on both SiO_2_/Si and TiO_2_/Si wafers, where the film on SiO_2_ appears slightly yellowish due to thin film interference. Considering the synthetic challenges of large‐scale thin‐film TMDs and high‐entropy nanomaterials [34, 35, 36], our approach provides a promising pathway toward scalable HETMD development.

### 2.2 Characterization of (MoWTaNbRu)S_2_ on TiO_2_/p‐Si Photocathodes

**FIGURE 2:**
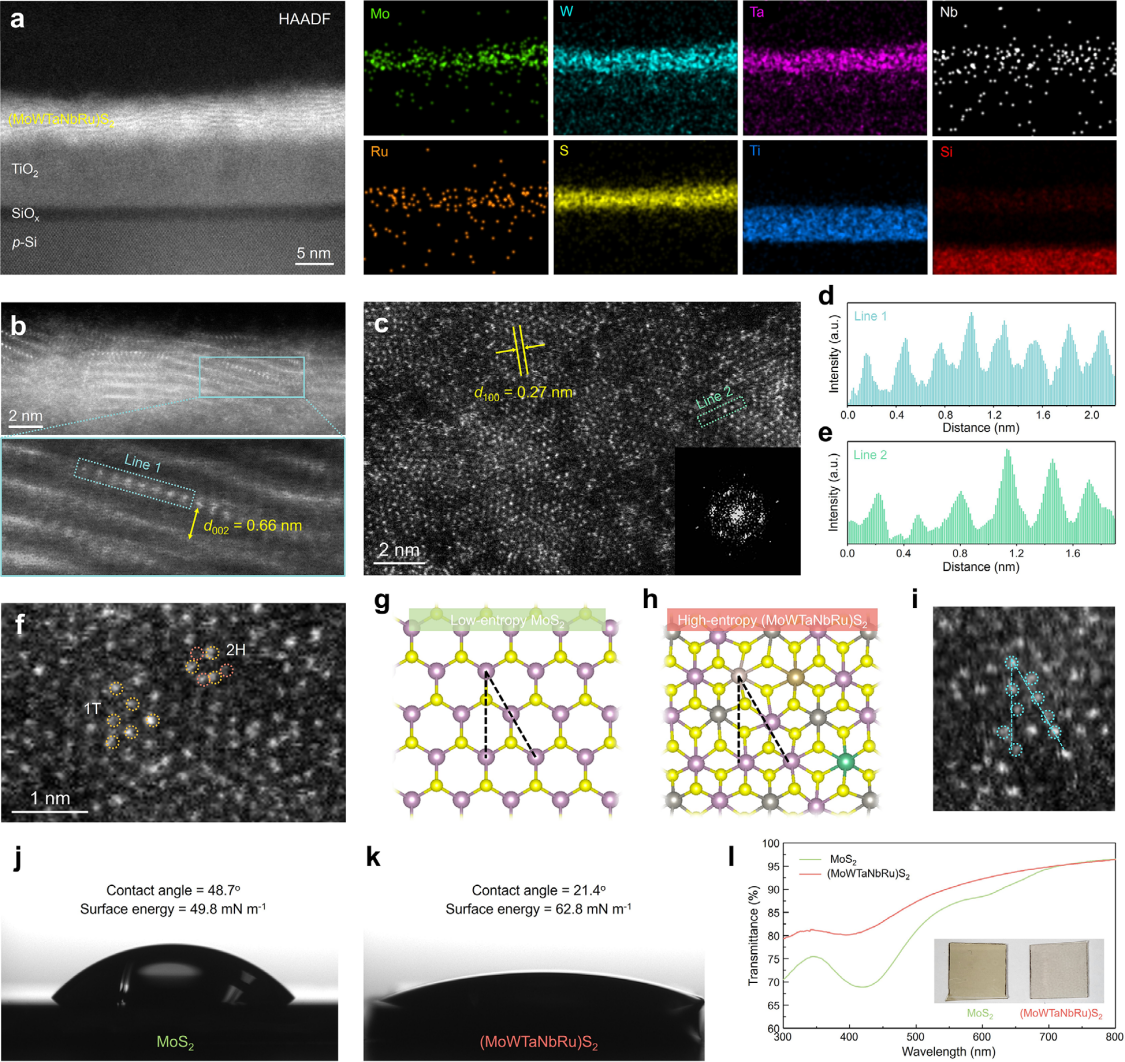
(a) Cross‐sectional HAADF‐STEM and EDS mapping images of (MoWTaNbRu)S_2_/TiO_2_/*p*‐Si photocathode. (b) High‐resolution cross‐sectional HAADF‐STEM image. (c) Top‐view HAADF‐STEM image and corresponding fast Fourier transformation pattern of (MoWTaNbRu)S_2_. (d,e) Intensity profiles along lines 1 and 2. (f) Magnified STEM image showing 2H and 1T phases of (MoWTaNbRu)S_2_ with lattice distortion. Atomic structure models of (g) 2H‐MoS_2_ and (h) 1T‐(MoWTaNbRu)S_2_ were constructed and optimized via DFT calculations. (i) Magnified STEM image of distorted 1T‐(MoWTaNbRu)S_2_. Contact angle images of (j) MoS_2_ and (k) (MoWTaNbRu)S_2_. (l) Optical transmittance of MoS_2_ and (MoWTaNbRu)S_2_.

The cross‐sectional high‐angle annular dark‐field scanning transmission electron microscopy (HAADF‐STEM) image and energy dispersive X‐ray spectroscopy (EDS) mappings in Figure 2a confirm a homogeneous distribution of Mo, W, Ta, Nb, Ru, and S elements within the (MoWTaNbRu)S_2_ film deposited on TiO_2_/*p*‐Si. Additional EDS mappings acquired from three representative locations across the 4‐inch wafer further confirm uniform elemental distribution, demonstrating consistent wafer‐scale compositional control of the high‐entropy TMD film (Figure S2). The elemental composition is quantified by inductively coupled plasma‐mass spectrometry (Table S2). In Figure 2b, the magnified cross‐sectional STEM images reveal a few layers of (MoWTaNbRu)S_2_ having (002) lattice fringes with an interlayer spacing of 0.66 nm. This interlayer distance is significantly larger than that of MoS_2_ (Figure S3), indicating an expanded van der Waals gap along the [001] direction, caused by electronic modulation and altered bonding angle within the crystal structure [37]. As shown in the top‐view STEM image and corresponding fast Fourier transformation pattern (Figure 2c), the lattice fringe spacing of 0.27 nm corresponds to the (100) plane of layered TMDs. Notably, atomic spots with varying brightness, arising from differences in atomic number among the constituent metals, provide clear evidence of atomic‐scale random distribution. The atomic intensity line profiles presented in Figure 2d,e, extracted from Figure 2b,c, reveal distinct intensity fluctuations at the transition metal sites, demonstrating random atomic dispersion.

As shown in the magnified STEM image in Figure 2f, both 2H and 1T phases coexist with distinct local lattice distortions. These distortions originate from varying atomic radii and asymmetric bonding environments, which induce local lattice strain and displace atoms from their average crystallographic positions [38, 39]. The atomic structure models of 2H‐MoS_2_, 1T‐(MoWRu)S_2_, and 1T‐(MoWTaNbRu)S_2_ are constructed and optimized via DFT calculations (Figure 2g,h; Figure S4). In (MoWTaNbRu)S_2_, most metal atoms exhibit noticeable deviations from their ideal crystallographic sites, whereas those in MoS_2_ and (MoWRu)S_2_ remain aligned. Notably, these simulated deviations are consistent with the atomic displacements observed in the HAADF‐STEM image (Figure 2i). Figures S5 and S6 show that (MoWTaNbRu)S_2_ exhibits curved morphology and atomic defects arising as a strain‐relief mechanism, allowing the lattice to accommodate distortions while preserving overall structure [40]. This is evidenced by a reduced water contact angle of 21.4°, compared to 48.7° for MoS_2_ and 23.8° for (MoWRu)S_2_ (Figure 2j,k; Figure S7 and Table S3). This superior hydrophilicity is expected to be critical for improving HER activity by facilitating electrolyte accessibility, enhancing mass transport, and promoting rapid H_2_ bubble detachment [41]. In terms of the optical characteristics, (MoWTaNbRu)S_2_ exhibits higher light transmittance than MoS_2_ in the wavelength range from 300 nm to 800 nm (Figure 2l; Figure S8) due to suppressed exciton absorption, which is advantageous for improving the light absorption of the underlying silicon photocathode.

### 2.3 Entropy‐Driven Local Electronic and Bonding Redistributions

**FIGURE 3:**
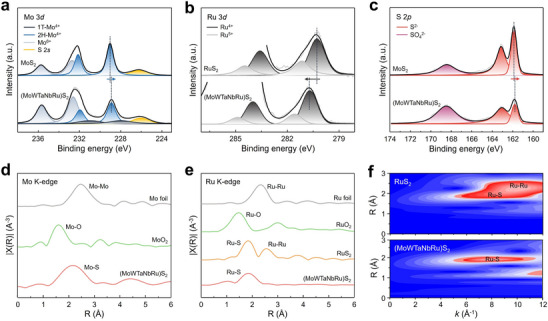
(a) Mo 3*d*, (b) Ru 3*d*, and (c) S 2*p* XPS spectra of (MoWTaNbRu)S_2_. (d) Mo K‐edge FT‐EXAFS spectra of Mo foil, MoO_2_, and (MoWTaNbRu)S_2_. (e) Ru K‐edge FT‐EXAFS spectra of Ru foil, RuO_2_, RuS_2_, and (MoWTaNbRu)S_2_. (f) The wavelet transforms (WT) for the k^3^‐weighted EXAFS signals of RuS_2_ and (MoWTaNbRu)S_2_.

We performed X‐ray photoelectron spectroscopy (XPS) on Mo 3*d*, W 4*f*, Ta 4*f*, Nb 3*d*, Ru 3*d*, and S 2*p* (Figure 3a–c; Figures S9–S14). Figure 3a presents the Mo 3*d* core level spectra of MoS_2_ and (MoWTaNbRu)S_2_, both showing spin‐orbit splitting doublets corresponding to Mo^4+^ in the 2H phase. Notably, (MoWTaNbRu)S_2_ exhibits additional peaks associated with the 1T phase [42], in agreement with the TEM observations. A slight negative shift in the 2H‐Mo^4+^ binding energy relative to MoS_2_ indicates an electron‐rich state of Mo. Similarly, the W^4+^ peaks in (MoWTaNbRu)S_2_ exhibit a negative binding energy shift compared to WS_2_, suggesting increased electron density at the W sites (Figure S10). Conversely, the Ru 3*d* and 3*p* peaks shift to higher binding energy relative to RuS_2_, implying an electron‐deficient state of Ru in (MoWTaNbRu)S_2_ (Figure 3b; Figure S11). In addition, the S^2−^ peaks in the S 2*p* spectra shift negatively relative to MoS_2_, indicating electron enrichment of S atoms (Figure 3c). Collectively, these binding energy shifts confirm entropy‐driven charge redistribution. Raman peak shifts with increasing metal incorporation provide additional evidence for this electronic redistribution (Figure S15).

Synchrotron‐based X‐ray absorption spectroscopy further elucidates the coordination environment. Mo and Ru K‐edge X‐ray absorption near‐edge structure (XANES) spectra (Figure S16) confirm oxidized states of Mo and Ru in (MoWTaNbRu)S_2_. The S K‐edge XANES spectra (Figure S17) show the lowest absorption edge energy for (MoWTaNbRu)S_2_, indicating electron enrichment of S atoms, consistent with XPS. The Mo K‐edge Fourier transformed X‐ray absorption fine structure (FT‐EXAFS) spectrum (Figure 3d) shows a dominant peak at 2.1 Å from Mo‐S bonds. Compared to Mo foil and MoO_2_, (MoWTaNbRu)S_2_ exhibits a broader peak, indicating a distribution of bond lengths and coordination rather than a single well‐defined bonding configuration. It suggests an increased degree of local structural disorder arising from lattice distortions. The Ru K‐edge FT‐EXAFS spectrum (Figure 3e) of RuS_2_ exhibits two noticeable peaks at 1.9 Å and 2.6 Å, corresponding to Ru‐S and Ru‐Ru coordination, respectively, reflecting its 3D atomic arrangement [43]. However, high‐entropy (MoWTaNbRu)S_2_ shows only a single Ru‐S peak, indicating Ru dispersion within a 2D TMD lattice. Further evidence is provided by the wavelet‐transformed EXAFS spectra of Ru K‐edge oscillations (Figure 3f), where only a single intensity maximum at 1.9 Å is observed for high‐entropy (MoWTaNbRu)S_2_ while two distinct intensity maxima are present in RuS_2_. Collectively, these results confirm the absence of second‐shell Ru‐Ru coordination and validate the incorporation of Ru into the layered TMD framework.

### 2.4 Photoelectrochemical HER Performance and Theoretical Investigations

**FIGURE 4:**
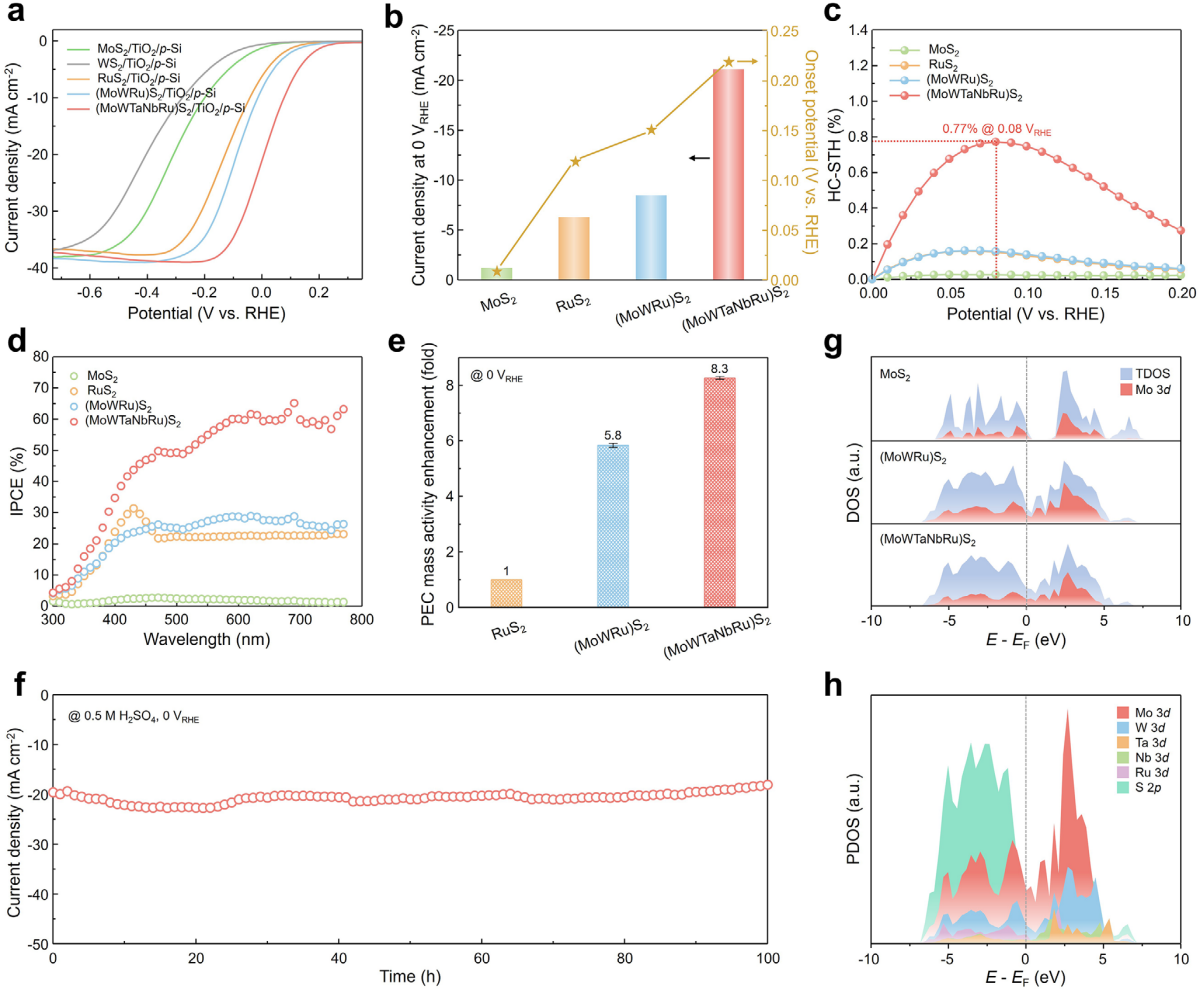
PEC‐HER performance and theoretical investigations. (a) Linear sweep voltammograms of the *p*‐Si photocathodes with MoS_2_, WS_2_, RuS_2_, (MoWRu)S_2_, and (MoWTaNbRu)S_2_ measured in 0.5 m H_2_SO_4_ electrolyte. (b) Comparison of the current density at 0 V_RHE_ and the onset potential. (c) Half‐cell solar‐to‐hydrogen (HC‐STH) energy conversion efficiencies. (d) Incident photon‐to‐current conversion efficiency (IPCE) measurements. (e) Comparison of photoelectrochemical mass activities among catalysts at 0 V_RHE_. Error bars represent the standard deviation. (f) Long‐term stability test over 100 h. (g) Total densities of states of MoS_2_, (MoWRu)S_2_, and (MoWTaNbRu)S_2_. (h) Partial densities of states of (MoWTaNbRu)S_2_.

The PEC‐HER performance was evaluated for (MoWTaNbRu)S_2_/TiO_2_/*p*‐Si photocathodes using a standard three‐electrode configuration with 0.5 m H_2_SO_4_ electrolyte under simulated AM 1.5 G solar illumination. We compared linear sweep voltammograms for Si photocathodes coated with MoS_2_, WS_2_, RuS_2_, medium‐entropy (MoWRu)S_2_, and high‐entropy (MoWTaNbRu)S_2_ (Figure 4a). The TiO_2_ passivation thickness, thin‐film catalyst thickness, and sulfurization temperature were optimized (Figures S18–S20). MoS_2_ and WS_2_ show poor PEC‐HER activity due to inert 2H basal planes. RuS_2_, known for favorable hydrogen binding, exhibits higher performance. The medium‐entropy (MoWRu)S_2_ yields a slight performance enhancement, arising from 1T phase formation. Notably, high‐entropy (MoWTaNbRu)S_2_ delivers the lowest onset potential and the highest photocurrent density. As summarized in Figure 4b and Table S4, (MoWTaNbRu)S_2_ achieved an onset potential of 0.22 V versus RHE and a photocurrent density of −21.1 mA cm^−2^ at 0 V versus RHE. The markedly lower apparent Tafel slope of (MoWTaNbRu)S_2_ (138 mV dec^−1^), relative to conventional MoS_2_ (232 mV dec^−1^) and WS_2_ (211 mV dec^−1^), reflects substantially accelerated reaction kinetics during PEC‐HER (Figure S21). Considering that TaS_2_ and NbS_2_ show poor PEC performance (Figure S22), the performance enhancement arises from the high‐entropy effect rather than a specific element. This high‐entropy design principle can also be extended to noble‐metal‐free TMD configurations, as (MoWTaNbNi)S_2_ and (MoWTaNbCo)S_2_ exhibit higher PEC‐HER performance than single‐metal TMDs (Figure S23). Moreover, (MoWTaNbRu)S_2_ outperforms Pt‐based catalysts such as metallic Pt and PtS_2_ films (Figure S24). The electrochemical measurements of catalysts deposited on carbon paper confirm that (MoWTaNbRu)S_2_ shows the highest intrinsic catalytic activity, lowest interfacial resistance, and largest electrochemically active surface area (Figures S25–S27).

Faradaic efficiency, measured by gas chromatography, reaches nearly 100% for (MoWTaNbRu)S_2_/TiO_2_/*p*‐Si, yielding high‐purity H_2_ (Figure S28). The photocathode achieved the highest maximum half‐cell solar‐to‐hydrogen efficiency of 0.77% (Figure 4c), reflecting a concurrent enhancement in both photocurrent density and photovoltage. Figure 4d presents the incident photon‐to‐current conversion efficiency (IPCE) spectra measured at 0 V versus RHE. Throughout the entire tested wavelength from 300 nm to 800 nm, (MoWTaNbRu)S_2_/TiO_2_/*p*‐Si achieves the highest IPCE, confirming the superior light harvesting. In Figure 4e, the PEC mass activity enhancement is calculated by normalizing the photocurrent densities to the Ru mass, as quantified by ICP‐MS (Table S5). The (MoWTaNbRu)S_2_ exhibits 8.2‐ and 1.4‐fold higher PEC mass activity compared to RuS_2_ and (MoWRu)S_2_, respectively, demonstrating that Ru utilization is greatly enhanced by the high‐entropy effect. The stability of photocathodes is evaluated by chronoamperometry at the applied potential of 0 V versus RHE (Figure 4f; Figure S29). The (MoWTaNbRu)S_2_/TiO_2_/*p*‐Si maintains its initial current density for 100 h, whereas the other photocathodes exhibit severe degradation. This exceptional stability arises from high‐entropy stabilization of the 1T phase, as further discussed in Figure 6.

DFT calculations were conducted to support the superior HER activity of (MoWTaNbRu)S_2_. Total density of states (Figure 4g) shows 2H‐MoS_2_ with a wide band gap and weak *d*‐orbital contributions at the Fermi level, while multi‐metal 1T‐TMDs introduce gap states and enhanced *d*‐orbital density at the Fermi level, facilitating charge transfer during surface reaction [44]. Partial density of states reveals strong 3*d* orbital contributions from Mo, W, Ta, Nb, and Ru across the Fermi level, whereas MoS_2_ and (MoWRu)S_2_ exhibit relatively smaller contributions from constituent metals (Figure 4h; Figure S30). The hydrogen adsorption Gibbs free energy (ΔG_H*_) is calculated for the top S sites of 2H‐MoS_2_ and 1T‐(MoWTaNbRu)S_2_ at *U* = 0 V versus RHE (Figure S31). The ΔG_H*_ of 2H‐MoS_2_ is 2.14 eV, suggesting that weak S‐H binding hinders H adsorption. However, the ΔG_H*_ of 1T‐(MoWTaNbRu)S_2_ is −0.18 eV, indicating an outstanding hydrogen adsorption and desorption capability.

### 2.5 Photoexcited Charge Transfer Kinetics

**FIGURE 5:**
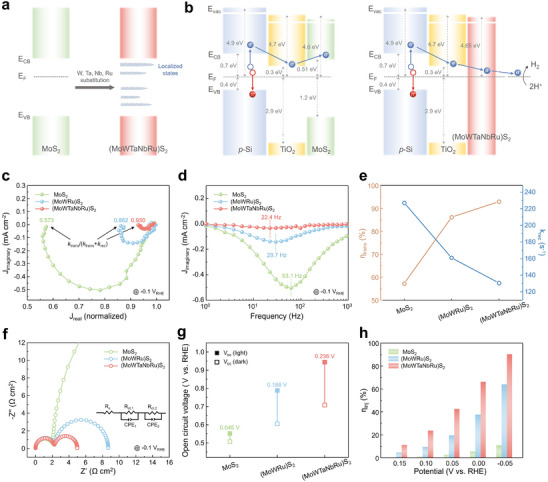
Photoexcited charge transfer kinetics. (a) Energy band schematics of MoS_2_ and high‐entropy (MoWTaNbRu)S_2_. (b) Energy band alignments of MoS_2_/TiO_2_/*p*‐Si and (MoWTaNbRu)S_2_/TiO_2_/*p*‐Si. (c) Intensity‐modulated photocurrent spectroscopy (IMPS) Nyquist plots at the applied potential of −0.1 V_RHE_. (d) Frequency‐dependent imaginary photocurrent plots. (e) Charge transfer efficiency and charge recombination constant. (f) Fitted photoelectrochemical impedance spectroscopy plots based on the equivalent circuit model shown in the inset. (g) Open circuit voltage measurements. (h) Surface charge injection efficiency.

Figure 5a compares band diagrams of pristine semiconducting MoS_2_ and high‐entropy (MoWTaNbRu)S_2_. Partial substitution of Mo^4+^ with W^4+^, Ta^5+^, Nb^5+^, and Ru^5+^ introduces additional electron donors, as represented by Ru 3*d* XPS analysis, resulting in the formation of localized states within the band gap. In addition, DFT calculations revealed continuous electronic states across the Fermi level, verifying its metallic character (Figure 4g). Based on these insights, we investigate photoinduced charge carrier energetics by determining electronic band structures (Figure 5b; Figure S32). Under steady‐state illumination, the energy difference between the conduction band minimum (E_CB_) and Fermi level (E_F_) in MoS_2_ (0.51 eV) is considerably larger than that in TiO_2_ (0.3 eV), resulting in energetically unfavorable electron transfer across the TiO_2_/MoS_2_ interface. However, metallic (MoWTaNbRu)S_2_ with a work function of 4.65 eV establishes a favorable alignment with TiO_2_, facilitating efficient migration of photogenerated electrons toward the electrolyte.

Intensity‐modulated photocurrent spectroscopy (IMPS) is performed to investigate the photogenerated charge carrier kinetics by analyzing charge transfer constant (k_trans_), charge recombination constant (k_rec_), and charge transfer efficiency (η_trans_). In the normalized IMPS spectra with the applied potential of −0.1 V versus RHE (Figure 5c; Figure S33a), k_trans_ / (k_trans_ + k_rec_) can be obtained from the ratio of real photocurrents at low‐frequency and high‐frequency intercepts. The sum of charge transfer and recombination constants, k_trans_ + k_rec_, is given by 2πf_max_, where f_max_ refers to the frequency at the maximum imaginary current in the frequency‐dependent imaginary photocurrent plot (Figure 5d; Figure S33b). The detailed calculations presented in the Supplementary Methods provide the values of k_trans_, k_rec_, and η_trans_. Figure 5e and Table S6 summarize the values of η_trans_ and k_rec_ for MoS_2_ (57.3 s^−1^, 227.2 s^−1^), (MoWRu)S_2_ (86.2 s^−1^, 25.8 s^−1^), and (MoWTaNbRu)S_2_ (93 s^−1^, 9.8 s^−1^). The (MoWTaNbRu)S_2_/TiO_2_/*p*‐Si photocathode exhibits the highest η_trans_ and lowest k_rec_, which is quantitative evidence for the improved electron transfer rate across the Helmholtz layer.

We analyze the interfacial charge transfer resistance (R_ct_) of photocathodes by photoelectrochemical impedance spectroscopy (PEIS) measurements (Figure 5f; Figure S34 and Table S7). The (MoWTaNbRu)S_2_/TiO_2_/*p*‐Si photocathode shows the lowest value of R_ct_, suggesting efficient electron transport to the H intermediate. It is notable that increasing the entropy in TMDs leads to a rise in η_trans_ and a simultaneous decrease in R_ct_, highlighting the high‐entropy‐driven acceleration of surface reaction kinetics (Figure S35). The open circuit voltage changes (ΔV_OC_) between dark and illuminated conditions (Figure 5g; Figures S36 and S37) are investigated to get insights into the driving force that the photoelectrodes provide for water reduction. The (MoWTaNbRu)S_2_/TiO_2_/*p*‐Si shows the largest ΔV_OC_ of 0.236 V, indicating that high‐entropy TMDs enhance both interfacial energetics and charge transfer kinetics at the semiconductor/electrolyte interface. We further evaluate the charge injection efficiency (η_inj_) by measuring *J–V* curves in an electrolyte containing 0.5 m K_3_Fe(CN)_6_ as an electron scavenger (Figure 5h; Figures S38 and S39). Compared to other samples, (MoWTaNbRu)S_2_ shows significantly high η_inj_ values at the whole potential range, and it reaches up to 90.3% at the potential of −0.05 V versus RHE, implying that high‐entropy alloying in TMDs markedly improves the separation of photoinduced electrons.

### 2.6 High‐Entropy‐Driven 1T Phase Stabilization

**FIGURE 6:**
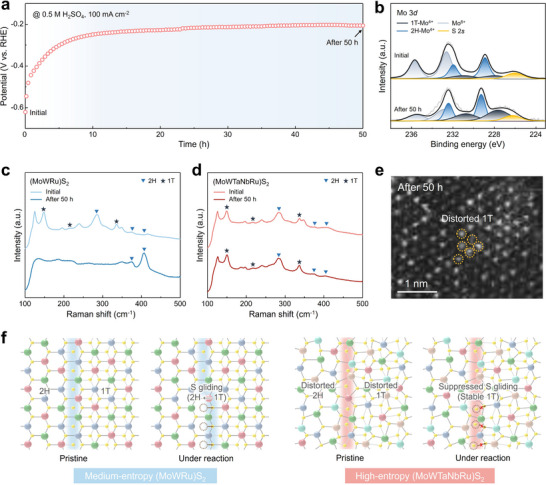
High‐entropy‐driven 1T phase stabilization. (a) Electrochemical stability test of (MoWTaNbRu)S_2_/carbon paper by chronopotentiometry at 100 mA cm^−2^ in 0.5 m H_2_SO_4_. (b) Mo 3*d* XPS spectra of (MoWTaNbRu)S_2_ before and after 50 h of reaction. Raman spectra of (c) medium‐entropy (MoWRu)S_2_ and (d) high‐entropy (MoWTaNbRu)S_2_ before and after 50 h of reaction. (e) HAADF‐STEM image of (MoWTaNbRu)S_2_ after 50 h of reaction. (f) Schematic atomic structures of (MoWRu)S_2_ and (MoWTaNbRu)S_2_ in the pristine state and under reaction, illustrating S gliding and phase stabilization, respectively.

Considering that the metallic 1T polymorph of TMDs is thermodynamically metastable relative to the semiconducting 2H phase [45], and that strong reduction potential can drive structural rearrangements from 1T to 2H, the superior stability of high‐entropy (MoWTaNbRu)S_2_ compared with single‐metal and medium‐entropy TMDs is noteworthy. To elucidate the intrinsic structural characteristics of the catalysts under electrochemical operation and uncover the origin of their robustness, we deposited them onto carbon paper and performed post‐reaction characterizations. As shown in Figure 6a, chronopotentiometry at 100 mA cm^−2^ in 0.5 m H_2_SO_4_ exhibits an initial decrease in overpotential within the first 10 h, followed by stable operation throughout the test. It is attributed to surface reconstruction during initial reaction, associated with the partial conversion of Mo^6+^ oxide species into Mo^4+^ [46]. Post‐reaction Mo 3*d* XPS spectra (Figure 6b) show a noticeable decrease in Mo^6+^ assigned to surface oxides, accompanied by increased contributions from both 1T‐ and 2H‐Mo^4+^ species. Notably, the critical point to highlight is the robustness of 1T phase in (MoWTaNbRu)S_2_ under prolonged operation. We further performed Raman spectroscopy to reveal the structural stability of (MoWTaNbRu)S_2_. The medium‐entropy (MoWRu)S_2_ initially exhibits both 1T and 2H peaks but completely converts to the 2H phase after 50 h of operation (Figure 6c). However, the high‐entropy (MoWTaNbRu)S_2_ preserves its original 1T peaks throughout the stability test (Figure 6d). Consistently, the HAADF‐STEM image after a 50 h stability test reveals the dominance of distorted 1T atomic structure, providing direct evidence of high‐entropy‐driven 1T phase stabilization (Figure 6e). As illustrated in Figure 6f, the 1T‐to‐2H structural rearrangement of conventional TMDs occurs by the sulfur plane gliding [47]. However, the structural distortion within high‐entropy TMDs introduces inhomogeneous activation barriers for atomic jumps in sulfur planes, lowering the overall diffusion rate [48]. Consequently, the 1T‐to‐2H structural transition is suppressed, leading to high stability of high‐entropy TMDs under electrochemical reactions. These findings highlight that high‐entropy‐driven sluggish atomic diffusion is critical for stabilizing the metastable 1T phase of TMDs.

## 3 Conclusion

In summary, we have developed a wafer‐scale, thermolysis‐driven high‐entropy (MoWTaNbRu)S_2_ thin‐film catalyst with a distorted 1T phase integrated onto a *p*‐Si photocathode for efficient and durable PEC hydrogen production. Entropy‐induced redistribution of local electronic states enhances the contribution of *d*‐orbitals at the Fermi level, which optimizes the hydrogen adsorption energetics. Photocarrier dynamics analyses confirm that the HETMD catalyst suppresses surface recombination and accelerates interfacial charge transport, leading to substantially improved PEC‐HER performance. Additionally, the 1T‐to‐2H phase transition observed in conventional TMDs under electrochemical operating conditions is fully suppressed due to sluggish S atom diffusion within the distorted lattice. As a result, the (MoWTaNbRu)S_2_/TiO_2_/*p*‐Si photocathode exhibits a remarkable photocurrent density of −21.1 mA cm^−2^ at 0 V versus RHE and outstanding durability for over 100 h. This study demonstrates how configurational entropy modulates photogenerated charge carrier dynamics and phase stability in 2D TMDs, offering new opportunities for next‐generation PEC devices.

## 4 Experimental Section

### 4.1 Synthesis of (MoWTaNbRu)S_2_ on TiO_2_/p‐Si

Boron‐doped (100) *p*‐type single crystal silicon wafers (1–10 Ω cm, DASOMRMS Co., Ltd) were cleaned in acetone, isopropyl alcohol, and DI water by ultrasonication for 15 min at each step and soaked in hydrofluoric acid for 30 s to remove the native SiO_2_ layer. The TiO_2_ layer with 8 nm thickness was deposited on a Si wafer by an electron beam evaporator (Korea Vacuum Tech Co, Ltd). The growth rate and pressure of the chamber were 0.08 Å s^−1^ and 1.0 × 10^−6^ mTorr, respectively. Then, the TiO_2_/*p*‐Si was treated by UV‐O_3_ for 30 min to make its surface highly hydrophilic.

A 2D high‐entropy (MoWTaNbRu)S_2_ thin film was deposited on TiO_2_/*p*‐Si via spin‐coating and thermal sulfurization of mixed metal precursors. Specifically, a mixture of 40 mmol L^−1^ (NH_4_)_2_MoS_4_ (Sigma‐Aldrich, 99.97% purity), 20 mmol L^−1^ (NH_4_)_2_WS_4_ (Sigma‐Aldrich, 99.9% purity), 10 mmol L^−1^ of TaCl_5_ (Sigma‐Aldrich, 99.8% purity), 10 mmol L^−1^ of NbCl_5_ (Sigma‐Aldrich, 99% purity), and 10 mmol L^−1^ of RuCl_3_·xH_2_O (Sigma‐Aldrich, 99.97% purity) dissolved in 5 mL ethylene glycol (Sigma‐Aldrich, 99.8% purity, anhydrous) was ultrasonicated for 6 h to ensure complete dissolution. The precursor solution was spin‐coated onto the TiO_2_/*p*‐Si substrate at 5000 rpm for 60 s. For the thermal sulfurization, we used a home‐built CVD system with the flow of N_2_/H_2_ (500/50 sccm) gases. The CVD furnace where the samples were located was heated to 550°C, followed by the sublimation of the sulfur powder (99.998%, Sigma‐Aldrich) at 300°C for 10 min. The transition metal ratios were controlled by adjusting the precursor molar ratios in the coating solution.

### 4.2 Synthesis of MoS_2_, WS_2_, TaS_2_, NbS_2_, RuS_2_, and (MoWRu)S_2_ on TiO_2_/p‐Si

A total of 40 mmol L^−1^ (NH_4_)_2_MoS_4_, 20 mmol L^−1^ (NH_4_)_2_WS_4_, 10 mmol L^−1^ TaCl_5_, 10 mmol L^−1^ NbCl_5_, and 10 mmol L^−1^ RuCl_3_·xH_2_O were dissolved in 5 mL ethylene glycol for MoS_2_, WS_2_, TaS_2_, NbS_2_, and RuS_2_, respectively. For (MoWRu)S_2_, 40 mmol L^−1^ (NH_4_)_2_MoS_4_, 20 mmol L^−1^ (NH_4_)_2_WS_4_, and 10 mmol L^−1^ RuCl_3_·xH_2_O were dissolved in ethylene glycol. Then, they were ultrasonicated for 6 h, followed by spin‐coating onto the TiO_2_/*p*‐Si at 5000 rpm for 60 s. Thermal sulfurization was conducted in the same way as the method of synthesizing (MoWTaNbRu)S_2_.

### 4.3 Characterizations

The TEM specimen of (MoWTaNbRu)S_2_/TiO_2_/*p*‐Si sample for the cross‐sectional image was prepared by focused ion beam (Helios G4, Thermo Fisher Scientific). The aberration‐corrected high‐angle annular dark field‐scanning TEM and energy‐dispersive X‐ray spectroscopy (EDS) mapping images were obtained using Cs corrected monochromated TEM (Themis Z, 200 kV). The semiangle of the probe‐forming aperture was 17.9 mrad. The inner and outer semiangles of the HAADF detector were ∼50 and 200 mrad. The probe current and dwelling time were 60 pA and 2 µs. The elemental composition and chemical states were explored by XPS (AXIS SUPRA, Kratos). The XPS peaks were fitted using XPSpeak41 software, and the backgrounds of the data were corrected by the Shirley method. The XAS analysis of Mo K‐edge, Ru K‐edge, and S K‐edge was conducted at the Pohang Light Source (PLS) in the Pohang Accelerator Laboratory (PAL), Republic of Korea. The Athena and Artemis in the Demeter software were used to transform and process the data. Raman spectroscopy was performed using a confocal microscope with an Ar laser operating at 532 nm (HEDA‐SERA, NOST). The atomic concentrations of constituent elements were measured by ICP‐MS (7900 ICP‐MS, Agilent Technologies). The transmittance and reflectance versus wavelength were measured by UV–vis spectroscopy (V‐770, JASCO). The contact angle of deionized water was measured by a contact angle analyzer (FEMTOFAB, Smart Drop). To calculate the surface energy, the Neumann model was applied. The Neumann's equation for the contact angle (θ) between a liquid and a solid surface is given by:

(1) $$\cos\;\theta =\;-1\;+\;2\;\sqrt{\frac{\gamma_{s}}{\gamma_{l}}}e^{-\beta {\left(\gamma_{s}-\gamma_{l}\right)}^{2}}$$

where θ represents the liquid–solid contact angle, *β* is a parameter related to the solid surface, γ_s_ is the surface energy of the solid, and γ_l_ is the surface energy of the liquid.

### 4.4 Photoelectrochemical measurements

A xenon arc lamp (Abet Technologies, LS150) was used as a light source, and the power intensity was calibrated to 100 mW cm^−2^ with a Si diode. For the (photo)electrochemical measurements, a three‐electrode system is used using a calomel electrode, graphite rod, and 0.5 m H_2_SO_4_ for reference electrode, counter electrode, and electrolyte, respectively. The electrochemical data were recorded on a potentiostat (Ivium Technologies, Nstat). For *I–V* curves, the potential was swept toward the cathodic direction at a scan rate of 10 mV s^−1^. All the potentials are converted into RHE according to the Nernst equation:

(2) $$E\left(\mathrm{RHE}\right)=E\left(\mathrm{SCE}\right)+E^{0}\left(\mathrm{SCE}\right)+0.059\;\times \;\mathrm{pH}$$

E(SCE) is the measured potential versus the calomel reference electrode, and E^0^(SCE) is 0.241 V at 25°C. The electrochemical double layer capacitance (C_dl_) was obtained by the CV curves at various scan rates from 20 to 120 mV s^−1^. The half‐cell solar‐to‐hydrogen conversion efficiency was calculated using the following equation:

(3) $$\mathrm{HC}-\;\text{STH}\;\left(\%\right)=\left[J\;\times \;\frac{V_{\mathrm{RHE}}-V_{H^{+}/H_{2}}}{P_{\mathrm{light}}}\right]\times 100\%$$

The incident photon‐to‐current conversion efficiency was measured using a monochromator (MonoRa150) with an applied potential of 0 V versus RHE. The stability of the photocathode was measured by chronoamperometry at a potential of 0 V versus RHE. To analyze the amount of the evolved H_2_ gas and calculate the Faradaic efficiency, the gas chromatography (Agilent GC 7890B) was used. The intensity‐modulated photocurrent spectroscopy was carried out using an electrochemical workstation (Zennium, Zahner) and a potentiostat (PP211, Zahner). The white light with 300 W m^−2^ intensity was illuminated, and the periodic modulation was swept with the frequency from 100 kHz to 1 Hz. The k_trans_ and k_rec_, and η_trans_ can be induced by equations given by:

(4) $$k_{\text{trans}}+k_{\text{rec}}=2\pi f_{\max}$$

(5) $$\eta_{\mathrm{trans}}=k_{\text{trans}}\;/\;\left(k_{\text{trans}}+k_{\text{rec}}\right)$$

where f_max_ is the frequency at which the imaginary photocurrent is at its maximum, and η_trans_ can be obtained by the ratio of real photocurrents at the low‐frequency and high‐frequency intercepts. Photoelectrochemical impedance spectroscopy was conducted with a frequency range from 500 kHz to 0.05 Hz at the potential of −0.1 V versus RHE. The PEIS spectra were fitted to the equivalent circuits using Z plot software. The open‐circuit potential change was determined by measuring the open‐circuit potential under dark and illuminated conditions. The injection efficiency (η_inj_) was calculated using the following equations:

(6) $$\eta_{inj}\;=\;\frac{J_{H_{2}O}}{J_{K_{3}Fe{\left(CN\right)}_{6}}}\;\;\;\times 100\%$$

where $J_{H_{2}O}$ and $J_{K_{3}Fe{\left(CN\right)}_{6}}$ are the photocurrent densities measured in 0.5 m H_2_SO_4_ without and with K_3_Fe(CN)_6_, respectively.

## Author Contributions

J.M., J.A.D., and H.W.J. supervised the project. S.E.J., J.H.S., and H.W.J. conceived the project. S.E.J. and J.H.S. developed the catalyst, performed the characterizations, and analyzed the experimental data. J.H.K. performed the theoretical simulations. H.L. measured IMPS. I.H.K, B.‐G.C., and S.K. helped to conduct XAS. W.S.C. performed gas chromatography measurements. S.K. helped to measure water contact angles. K.C.K. helped to synthesize the catalyst. C.‐H.L., J.P., and J.A.D. contributed to the manuscript editing. The manuscript was mainly written by S.E.J., J.H.S., J.A.D., and H.W.J. All authors discussed the results and commented on the manuscript at all stages.

## Conflicts of Interest

The author declares no conflict of interest.

## Supporting information




**Supporting File**: adma73236‐sup‐0001‐SuppMat.docx.

## Data Availability

The data that support the findings of this study are available from the corresponding author upon reasonable request.
