# Supplementary material for: Wafer‐Scale 2D High‐Entropy Transition Metal Dichalcogenide Thin‐Film Catalysts for Efficient and Durable Photoelectrochemical Hydrogen Production
Source: Adv Mater. 2026 May 6;38(32):e73236. doi: 10.1002/adma.73236 (PMC13244812; doi:10.1002/adma.73236)
Supplement: Supplementary file 1 — Supporting File: adma73236‐sup‐0001‐SuppMat.docx. [file ADMA-38-e73236-s001.docx]

Supplementary Information

**Wafer-Scale 2D High-Entropy Transition Metal Dichalcogenide Thin-Film Catalysts for Efficient and Durable Photoelectrochemical Hydrogen Production**

*Sang Eon Jun*^†^*, Jin Ho Seo*^†^*, Jaehyun Kim, Hyungsoo Lee, Seongbeen Kim, Woo Seok Cheon, Sabina Kim, In Hye Kwak, Byeong-Gwan Cho, Ki Chang Kwon, Chul-Ho Lee, Jungwon Park, Jooho Moon,^*^ Jennifer A. Dionne,^*^ Ho Won Jang^*^*

S. E. Jun, J. H. Seo, J. Kim, W. S. Cheon, S. Kim, H. W. Jang

Department of Materials Science and Engineering, Research Institute of Advanced Materials, Seoul National University, Seoul 08826, Korea

E-mail: hwjang@snu.ac.kr

S. E. Jun, J. A. Dionne

Department of Materials Science and Engineering, Stanford University, Stanford, CA, USA

E-mail: jdionne@stanford.edu

H. Lee, J. Moon

Department of Materials Science and Engineering, Yonsei University, Seoul 03722, Republic of Korea

E-mail: jmoon@yonsei.ac.kr

S. Kim

Department of Chemical and Biomolecular Engineering, Korea Advanced Institute of Science and Technology (KAIST), Daejeon 34141, Republic of Korea

I. H. Kwak, B.-G Cho

Korea Basic Science Institute, Daejeon 34133, Republic of Korea

K. C. Kwon

Division of Chemical and Material Metrology, Korea Research Institute of Standards and Science (KRISS), Daejeon 34133, Republic of Korea

Department of Applied Measurement Science, University of Science and Technology (UST), Daejeon 34113, Republic of Korea

C.-H. Lee

Department of Electrical and Computer Engineering, Seoul National University, Seoul 08826, Republic of Korea

Inter-university Semiconductor Research Center, Seoul National University, Seoul 08826, Republic of Korea

J. Park

School of Chemical and Biological Engineering, Institute of Chemical Processes, Seoul National University (SNU), Seoul 08826 Republic of Korea

Center for Nanoparticle Research, Institute for Basic Science (IBS), Seoul 08826, Republic of Korea

Institute of Engineering Research, College of Engineering, Seoul National University, Seoul 08826, Republic of Korea

Advanced Institutes of Convergence Technology, Seoul National University, Gyeonggi-do 16229, Republic of Korea

J. A. Dionne

Department of Radiology, Stanford University, Stanford, CA, USA

H. W. Jang

Advanced Institutes of Convergence Technology, Seoul National University, Gyeonggi-do 16229, Republic of Korea

†These authors contributed equally to this work

**CONTENTS**

**Supplementary Figures**

**Figure S1 |** Photographic image of (MoWTaNbRu)S_2_ synthesized on a 4-inch Si wafer.

**Figure S2 |** Cross-sectional EDS mapping images at different sites of 4-inch wafer-scale (MoWTaNbRu)S_2_/TiO_2_/*p*-Si.

**Figure S3 |** Cross-sectional HAADF-STEM image of MoS_2_.

**Figure S4 |** Atomic structure model of 1T-(MoWRu)S_2_ and 2D coordination bonding angles between transition metals and their neighboring metal atoms.

**Figure S5 |** Cross-sectional HR-TEM images of MoS_2_ and (MoWTaNbRu)S_2_.

**Figure S6 |** HR-TEM image of (MoWTaNbRu)S_2_ with atomic defects.

**Figure S7 |** Water contact angles of RuS_2_ and (MoWRu)S_2_. Contact angles and calculated surface energies of MoS_2_, RuS_2_, (MoWRu)S_2_, and (MoWTaNbRu)S_2_.

**Figure S8 |** Absorbance and reflectance versus wavelength spectra of MoS_2_ and (MoWTaNbRu)S_2_.

**Figure S9 |** XPS wide scans of MoS_2_/TiO_2_/*p*-Si, RuS_2_/TiO_2_/*p*-Si, (MoWRu)S_2_/TiO_2_/*p*-Si, and (MoWTaNbRu)S_2_/TiO_2_/*p*-Si.

**Figure S10 |** W 4*f* XPS spectra of WS_2_ and (MoWTaNbRu)S_2_.

**Figure S11 |** Ru 3*p* XPS spectra of RuS_2_ and (MoWTaNbRu)S_2_.

**Figure S12 |** Ta 4*f* and Nb 3*d* XPS spectra of (MoWTaNbRu)S_2_/TiO_2_/*p*-Si.

**Figure S13 |** S 2*p* XPS spectra of RuS_2_.

**Figure S14 |** Mo 3*d*, W 4*f*, Ru 3*d*, and S 2*p* XPS spectra of (MoWRu)S_2_.

**Figure S15 |** Raman spectra of MoS_2_, WS_2_, (MoWRu)S_2_, and (MoWTaNbRu)S_2_.

**Figure S16 |** Mo and Ru K-edge XANES spectra.

**Figure S17 |** S K-edge XANES spectra of MoS_2_, (MoWRu)S_2_, and (MoWTaNbRu)S_2_.

**Figure S18 |** LSV curves for PEC-HER with varying thicknesses of the TiO_2_ passivation layer.

**Figure S19 |** LSV curves for PEC-HER with varying the rotating speed during spin-coating of precursor solution for high-entropy (MoWTaNbRu)S_2_.

**Figure S20 |** LSV curves for PEC-HER with varying sulfurization temperatures.

**Figure S21 |** Tafel plots of the photocathodes with MoS_2_, WS_2_, RuS_2_, (MoWRu)S_2_, and (MoWTaNbRu)S_2_.

**Figure S22 |** LSV and PEIS plots of the photocathodes with TaS_2_ and NbS_2_ in 0.5 M H_2_SO_4_ electrolyte.

**Figure S23 |** LSV curves of photocathodes with (MoWTaNbNi)S_2_, (MoWTaNbCo)S_2_, and single-metal TMDs.

**Figure S24 |** LSV curves of photocathodes with Pt, PtS_2_, and (MoWTaNbRu)S_2_ thin-film catalysts.

**Figure S25 |** Electrochemical measurements of TMDs deposited on carbon paper.

**Figure S26 |** Cyclic voltammogram curves recorded at different scan rates and electrochemical double-layer capacitances of MoS_2_, RuS_2_, (MoWRu)S_2_, and (MoWTaNbRu)S_2_.

**Figure S27 |** Cyclic voltammogram curves recorded at different scan rates and electrochemical double-layer capacitances of WS_2_, TaS_2_, and NbS_2_.

**Figure S28 |** Faradaic efficiency and evolved hydrogen gas of (MoWTaNbRu)S_2_ during PEC-HER.

**Figure S29 |** Photoelectrochemical stability test of MoS_2_, RuS_2_, and (MoWRu)S_2_ by chronoamperometry at the applied potential of 0 V versus RHE.

**Figure S30 |** Partial density of states (PDOSs) of MoS_2_ and (MoWRu)S_2_.

**Figure S31 |** Atomic models of 2H-MoS_2_ and 1T-(MoWTaNbRu)S_2_ with H adsorption. Hydrogen adsorption Gibbs free energies (ΔG_H*_) of 2H-MoS_2_ and 1T-(MoWTaNbRu)S_2_ calculated for the top S sites at U = 0 V versus RHE.

**Figure S32 |** UPS and XPS valence-band spectra as a function of binding energy.

**Figure S33 |** Intensity-modulated photocurrent spectroscopy (IMPS) Nyquist plot and frequency dependent imaginary photocurrent plot of RuS_2_/TiO_2_/*p*-Si at the applied potential of -0.1 V versus RHE.

**Figure S34 |** Photoelectrochemical impedance spectroscopy plot of RuS_2_/TiO_2_/*p*-Si.

**Figure S35 |** Charge transfer efficiencies obtained from IMPS and charge transfer resistances at the interfaces obtained from PEIS of MoS_2_, (MoWRu)S_2_, and (MoWTaNbRu)S_2_.

**Figure S36 |** Open circuit potential measurements in the dark condition and under illumination.

**Figure S37 |** Open circuit potential difference of RuS_2_/TiO_2_/*p*-Si.

**Figure S38 |** J-V curves of photocathodes measured in 0.5 M H_2_SO_4_ + 0.5 M K_3_Fe(CN)_6_ electrolyte.

**Figure S39 |** Injection efficiency of RuS_2_/TiO_2_/*p*-Si.

**Supplementary Tables**

**Table S1 |** Synthesis of recently reported high-entropy transition metal chalcogenides.

**Table S2 |** Inductively coupled plasma-mass spectrometry (ICP-MS) measurement data.

**Table S3 |** Contact angles and surface energies of photocathodes.

**Table S4 |** Onset potentials and current densities at 0 V versus RHE of photocathodes.

**Table S5 |** Ru concentration measured by inductively coupled plasma-mass spectrometry (ICP-MS).

**Table S6 |** Frequency at the maximum imaginary current, charge transfer efficiency, charge transfer constant, and charge recombination constant.

**Table S7 |** Fitted interfacial charge transport resistances obtained by PEIS.

**Supplementary Methods**

**DFT calculations**

**Calculations of k_trans_ and k_rec_**

**Supplementary References**

**Supplementary Figures**


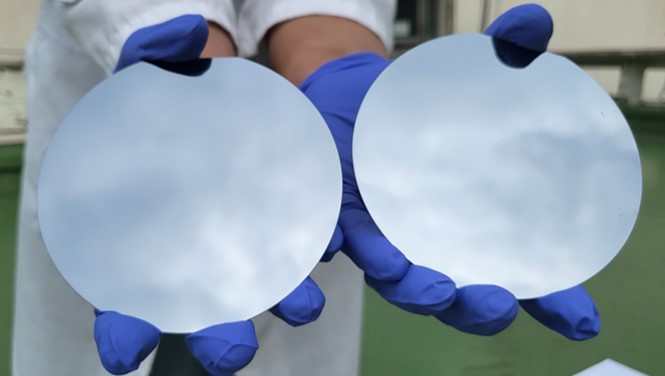


**Figure S1 |** Photographic image of (MoWTaNbRu)S_2_ synthesized on a 4-inch TiO_2_/Si wafer (Left: bare *p*-Si, Right: (MoWTaNbRu)S_2_/TiO_2_/*p*-Si).


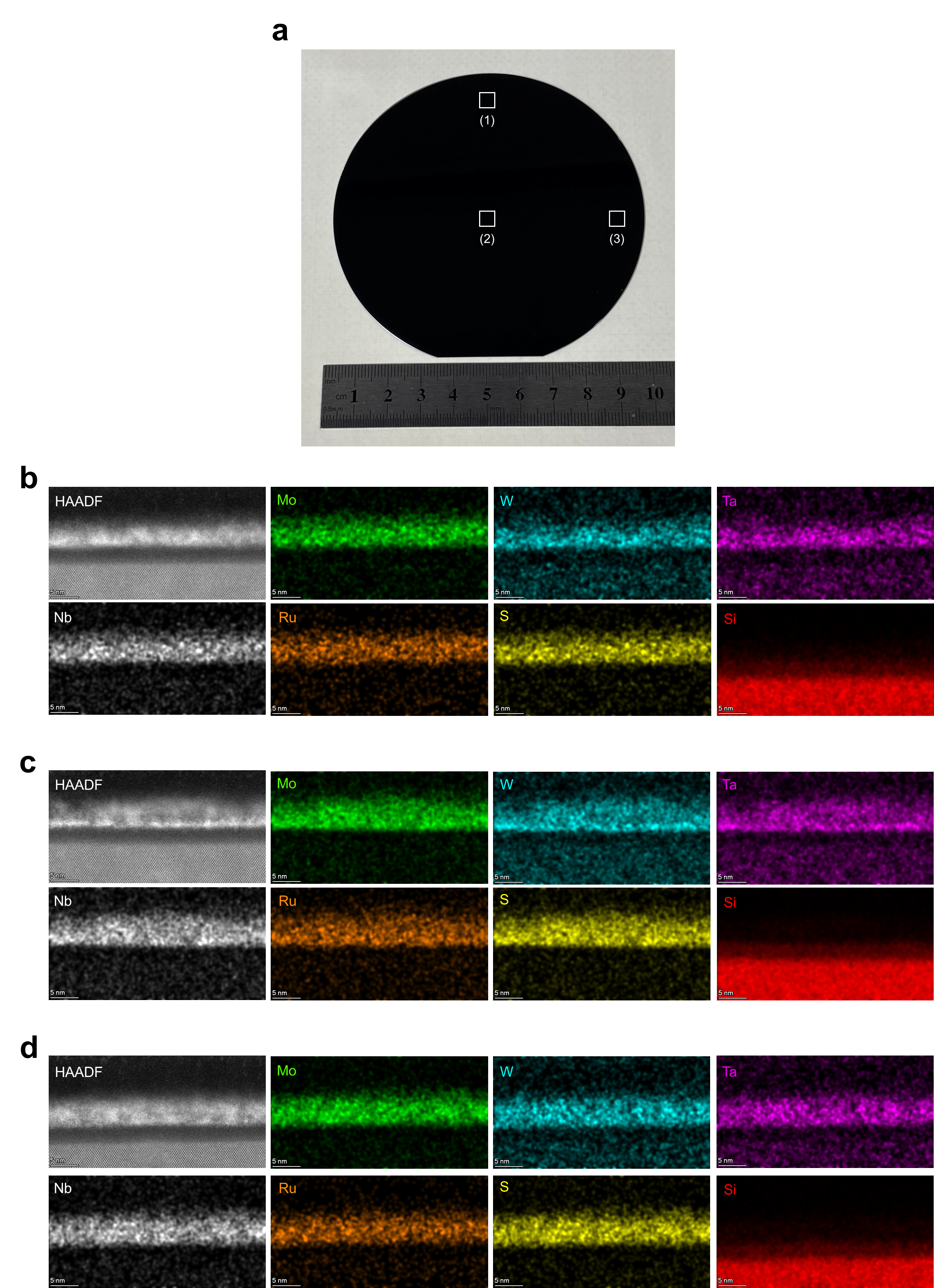


**Figure S2 |** (a) Photographic image of 4-inch wafer-scale (MoWTaNbRu)S_2_/TiO_2_/*p*-Si photocathode. Cross-sectional EDS mapping images at site (b) (1), (c) (2), and (d) (3).

**
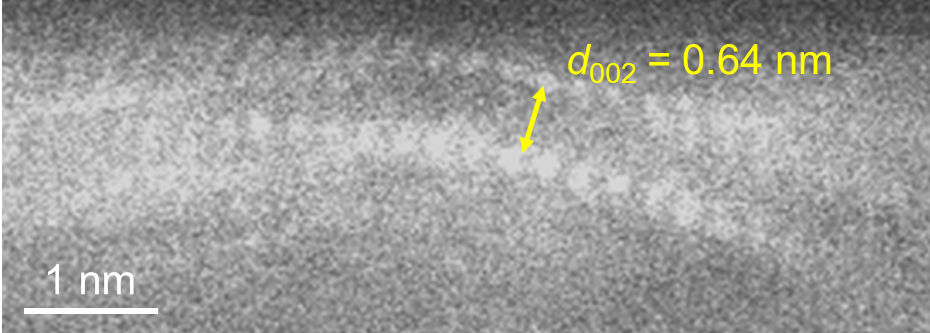
**

**Figure S3 |** Cross-sectional HAADF-STEM image of MoS_2_.

**
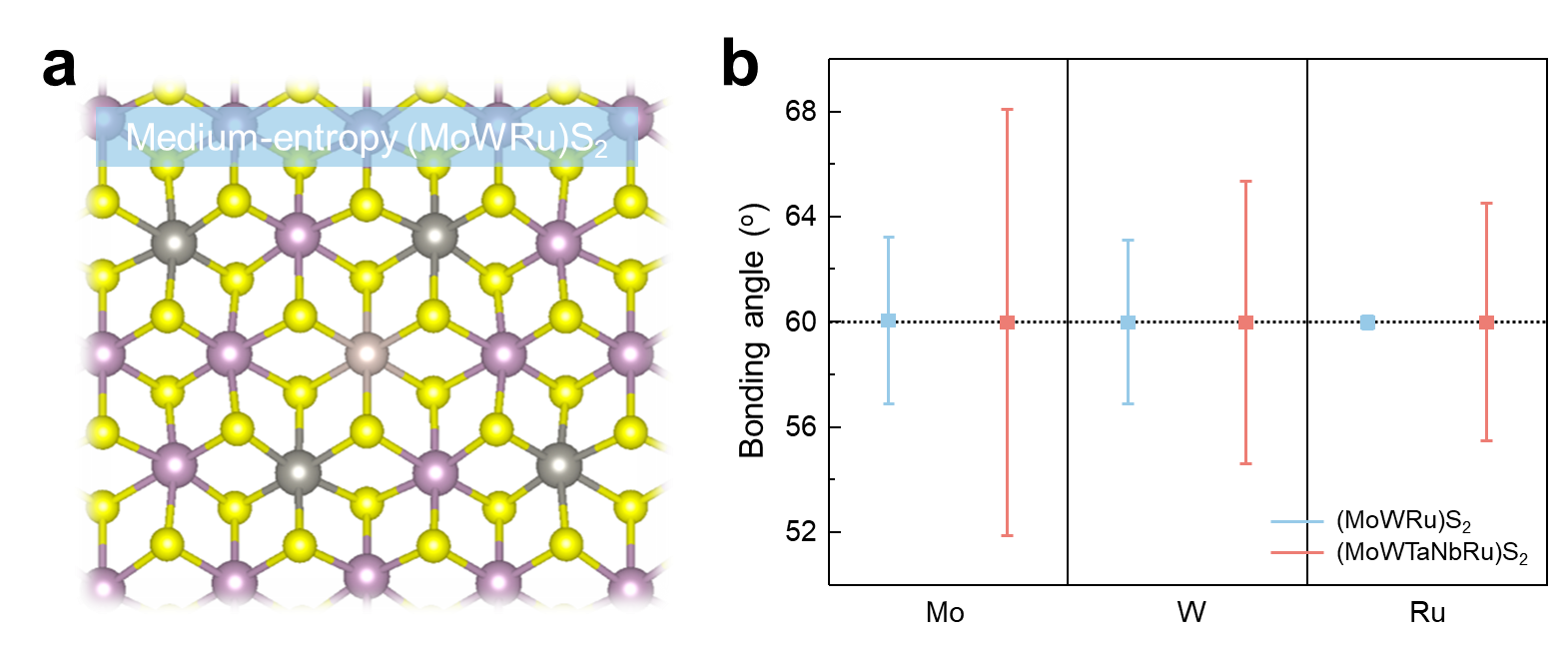
**

**Figure S4 |** (a) Atomic structure model of 1T-(MoWRu)S_2_ constructed and optimized via DFT calculations. (b) 2D coordination bonding angles between transition metals and their neighboring metal atoms.

**
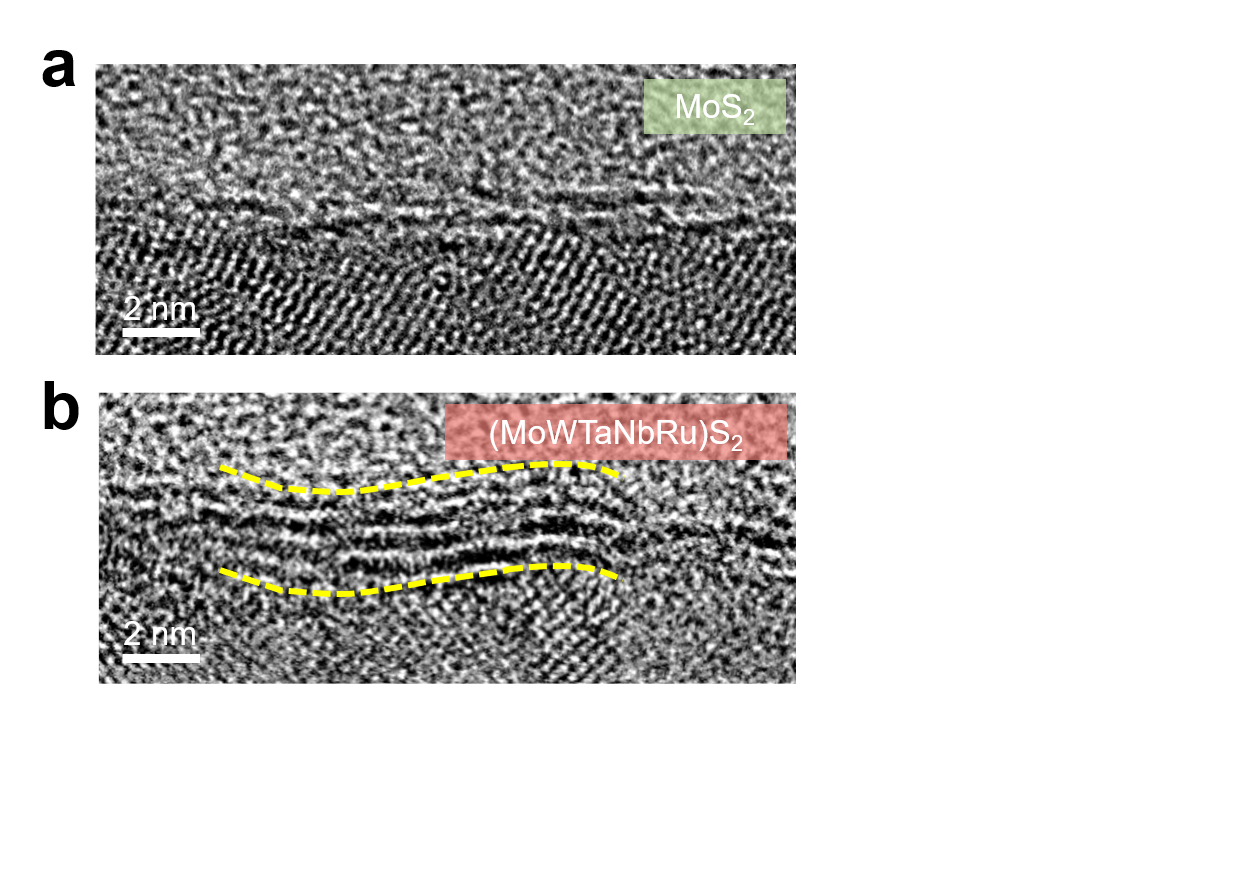
**

**Figure S5 |** Cross-sectional HR-TEM images of (a) MoS_2_ and (b) (MoWTaNbRu)S_2_.

Cross-sectional HR-TEM images (Figure S5) reveal distinct morphological differences between MoS_2_ and high-entropy (MoWTaNbRu)S_2_. MoS_2_ shows an atomically flat surface while (MoWTaNbRu)S_2_ exhibits an atomically curved morphology with increased surface roughness.

**
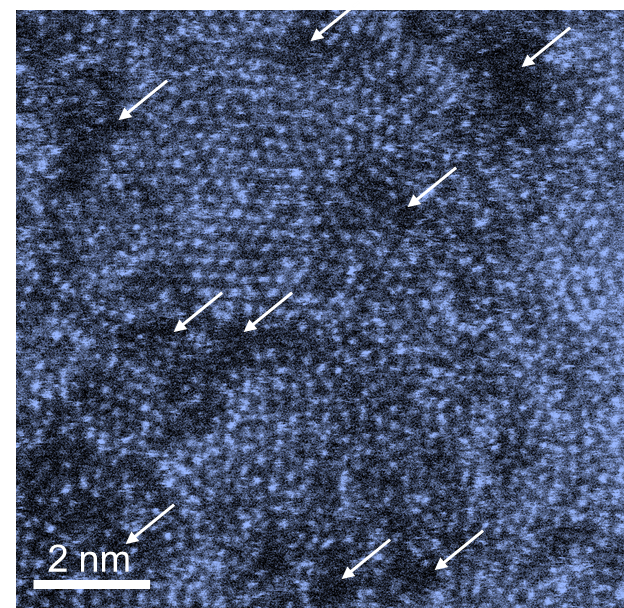
**

**Figure S6 |** HR-TEM image of (MoWTaNbRu)S_2_ with atomic defects.

**
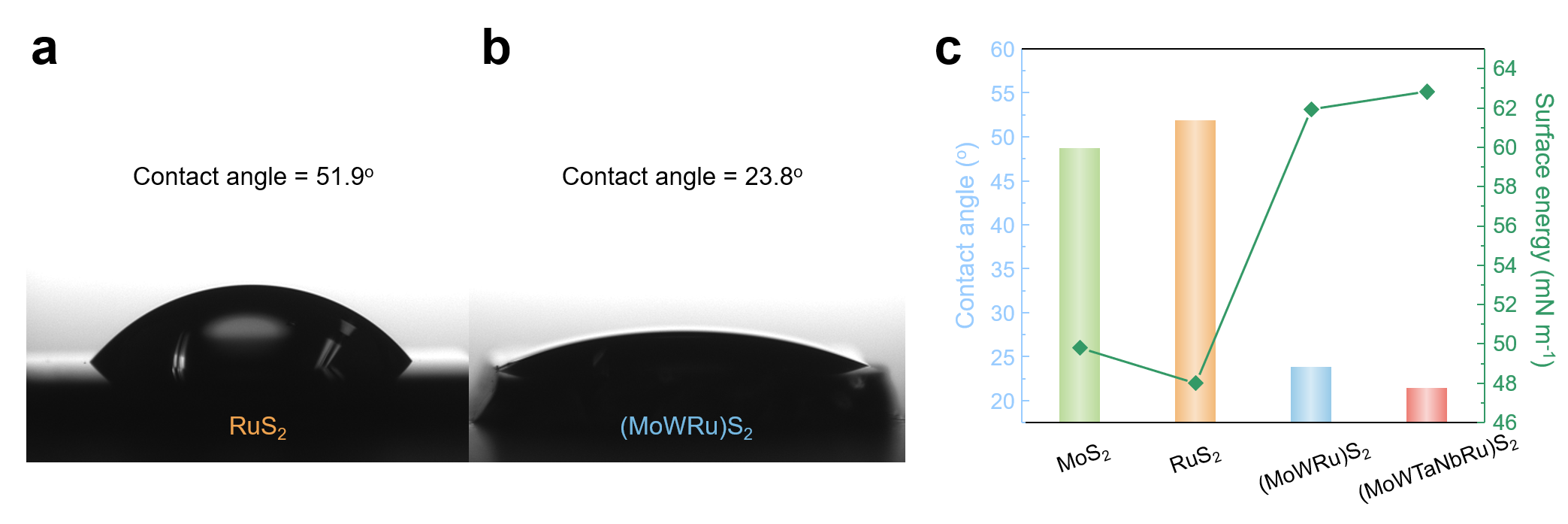
**

**Figure S7 |** Water contact angles of (a) RuS_2_ and (b) (MoWRu)S_2_. (c) Contact angles and calculated surface energies of MoS_2_, RuS_2_, (MoWRu)S_2_, and (MoWTaNbRu)S_2_.


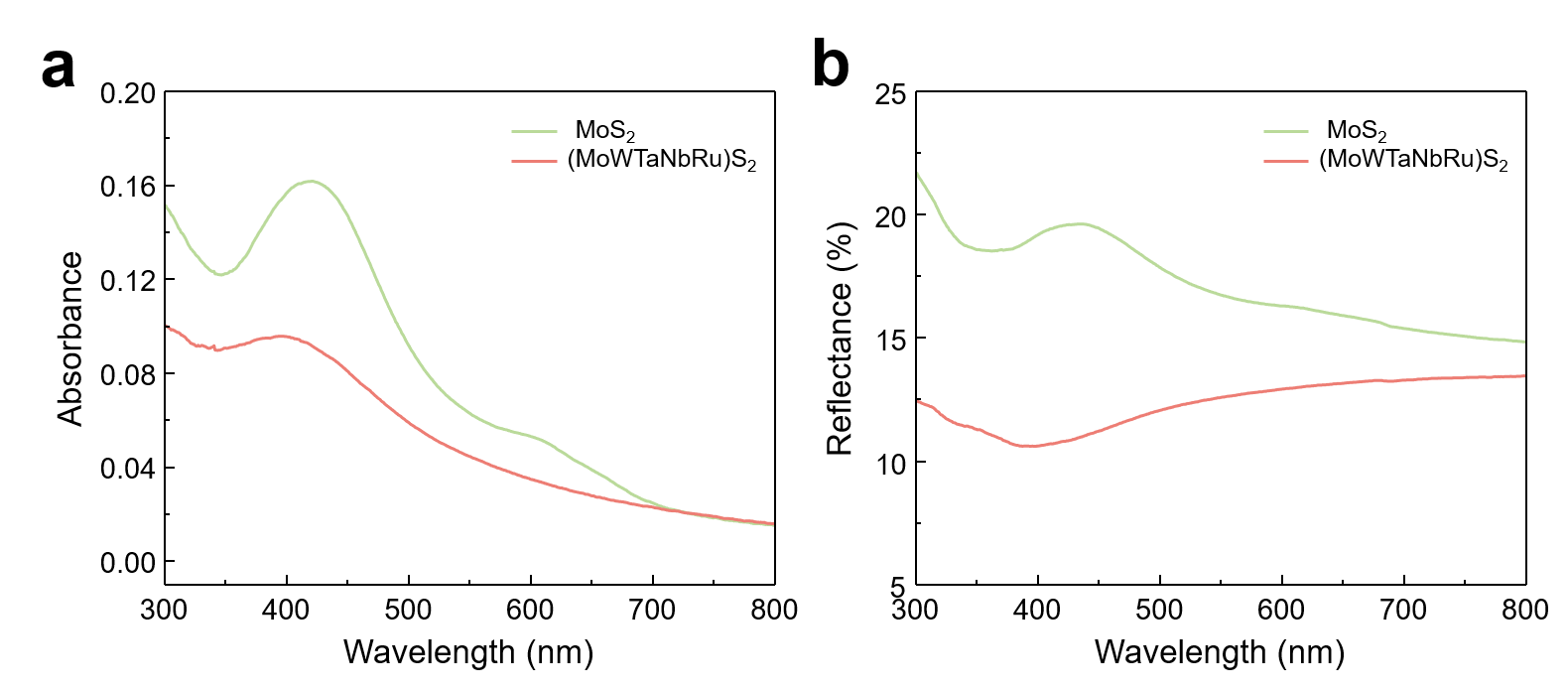


**Figure S8 |** (a) Absorbance and (b) reflectance versus wavelength spectra of MoS_2_ and (MoWTaNbRu)S_2_.


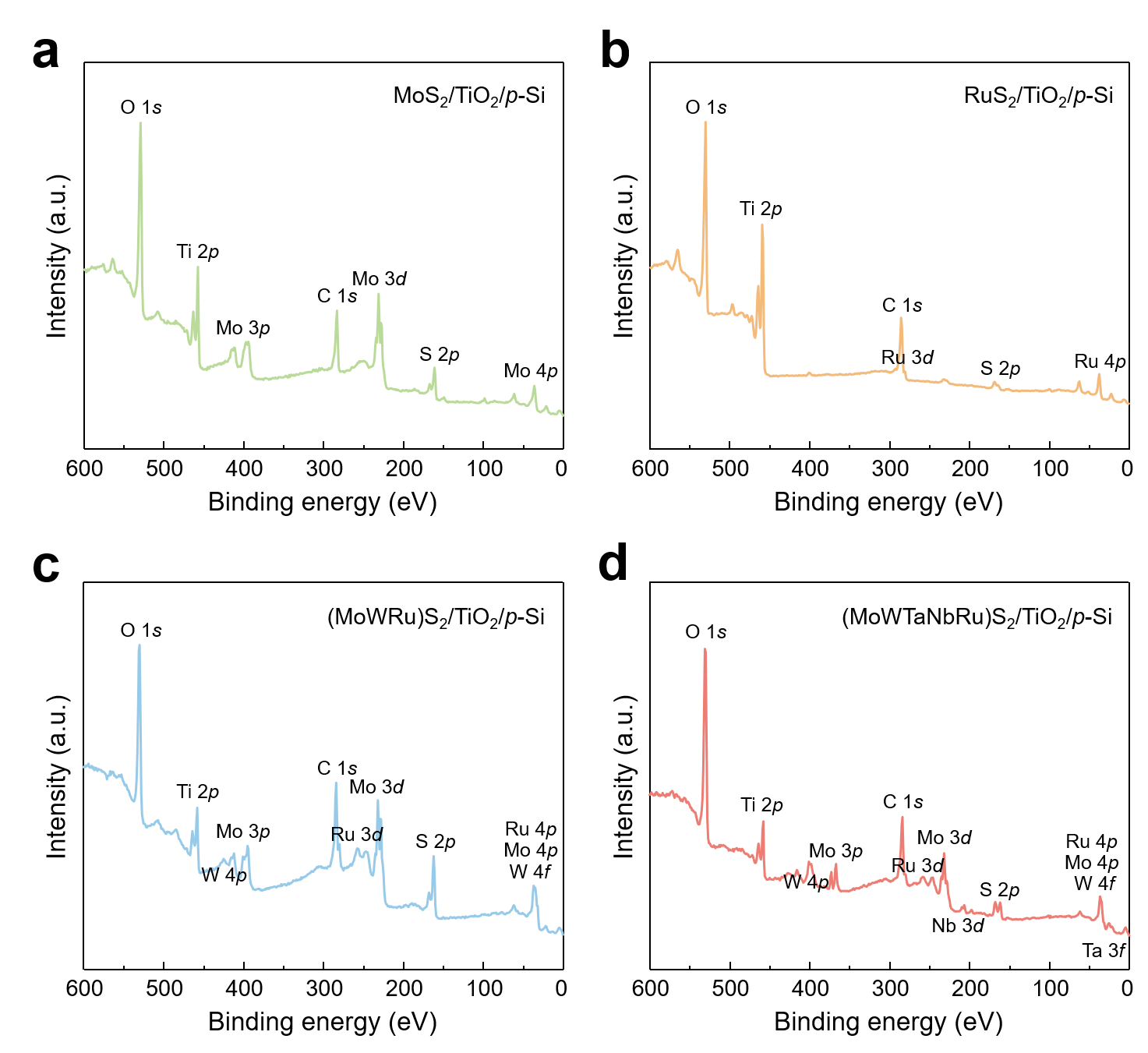


**Figure S9 |** XPS wide scans of (a) MoS_2_/TiO_2_/*p*-Si, (b) RuS_2_/TiO_2_/*p*-Si, (c) (MoWRu)S_2_/TiO_2_/*p*-Si, and (d) (MoWTaNbRu)S_2_/TiO_2_/*p*-Si.


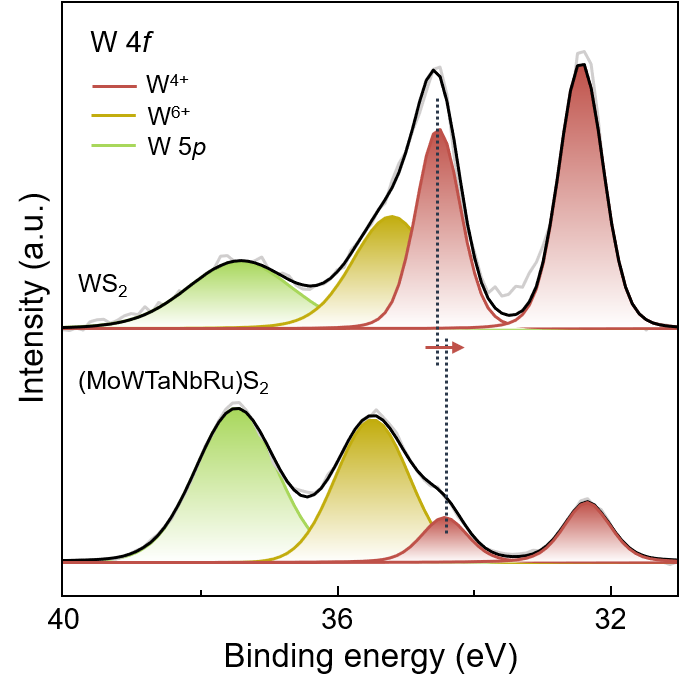


**Figure S10 |** W 4*f* XPS spectra of WS_2_ and (MoWTaNbRu)S_2_.


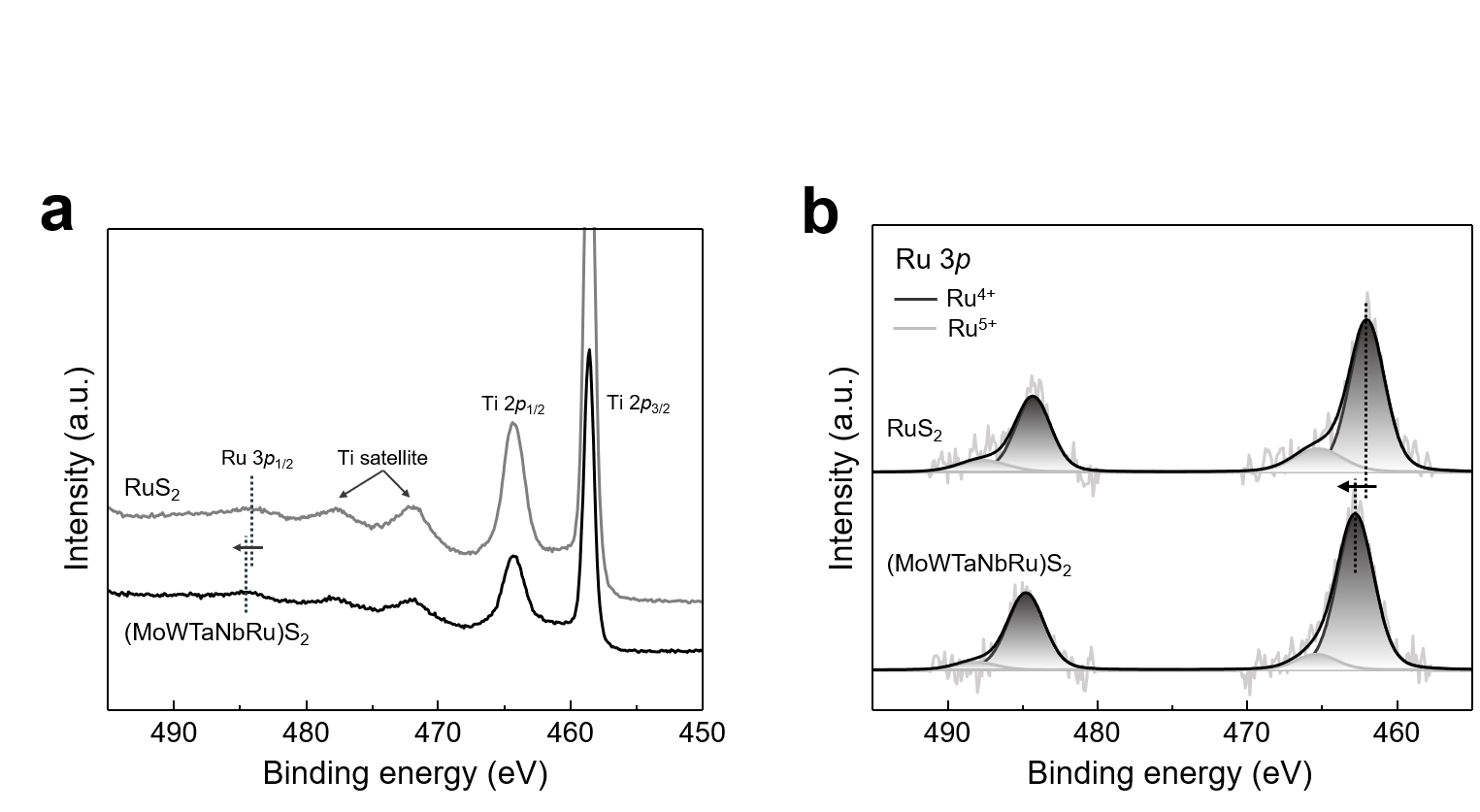


**Figure S11 |** Ru 3*p* XPS spectra of RuS_2_ and (MoWTaNbRu)S_2_ on (a) TiO_2_/*p*-Si and (b) SiO_2_/Si substrates.


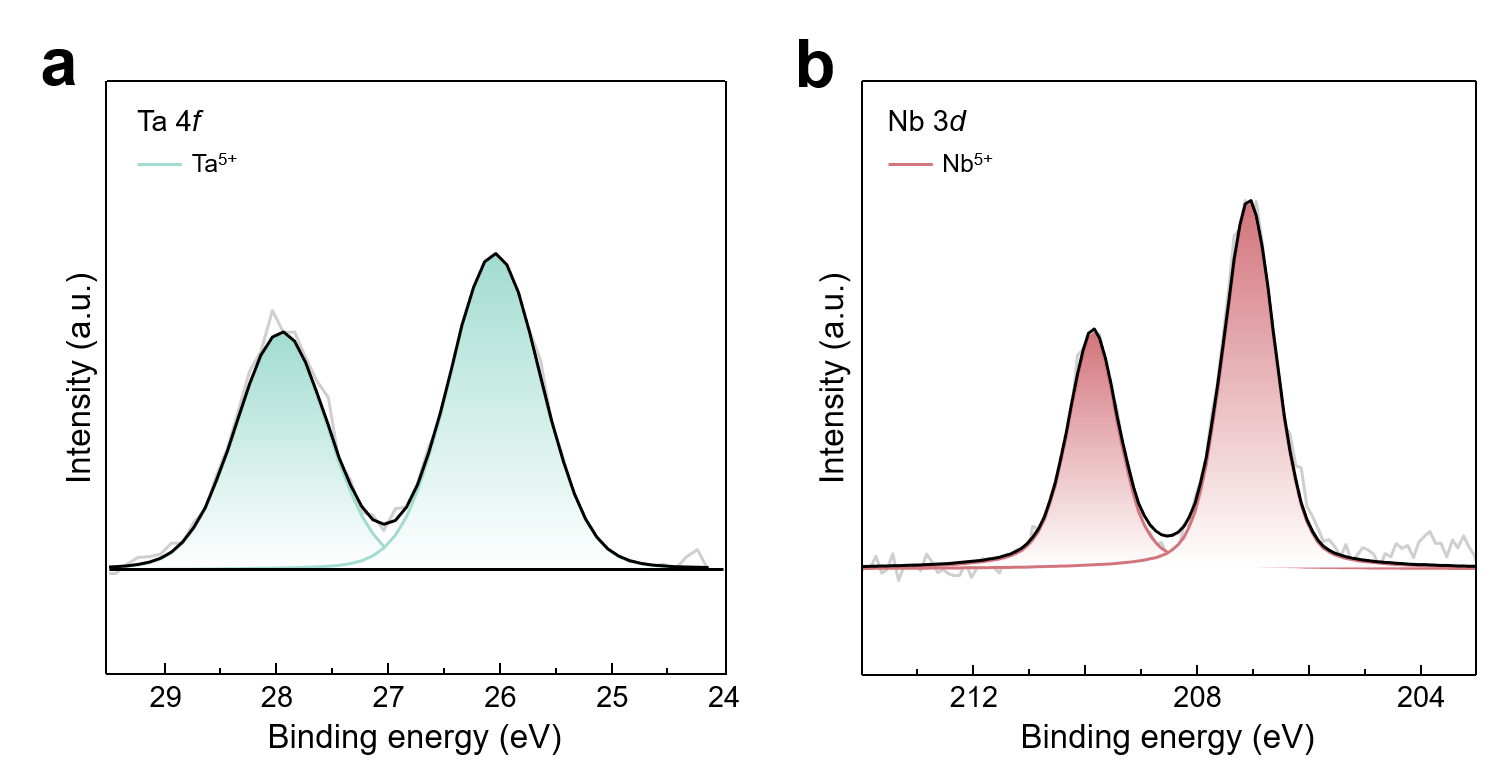


**Figure S12 |** (a) Ta 4*f* and (b) Nb 3*d* XPS spectra of (MoWTaNbRu)S_2_.

Both Ta and Nb exhibit 5+ oxidation states, which are more oxidized than the 4+ states typically reported for TaS_2_ and NbS_2_.^1,2^ This suggests that Ta and Nb adopt relatively electron-deficient states within the high-entropy lattice, consistent with the behavior observed for Ru.


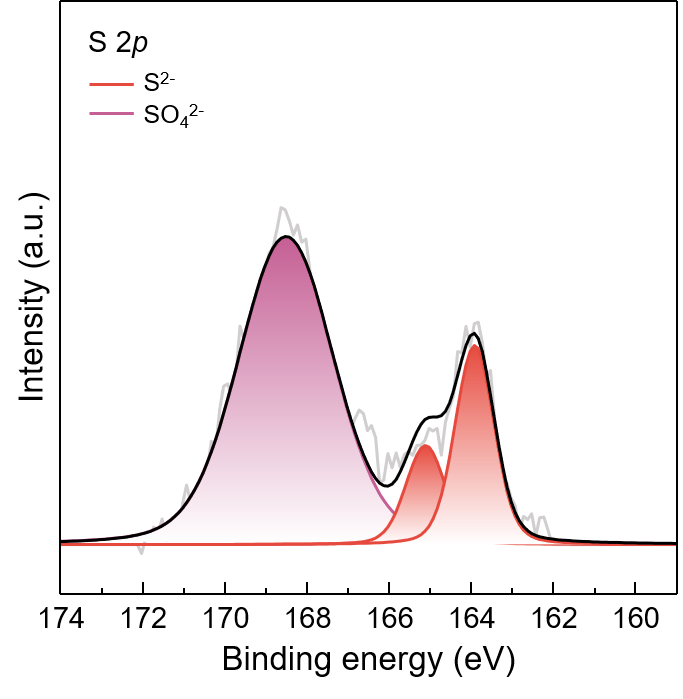


**Figure S13 |** S 2*p* XPS spectra of RuS_2_.

The binding energies of S 2*p* peaks in (MoWTaNbRu)S_2_ is higher than that in RuS_2_, indicating the absence of 3D Ru-S bonding.


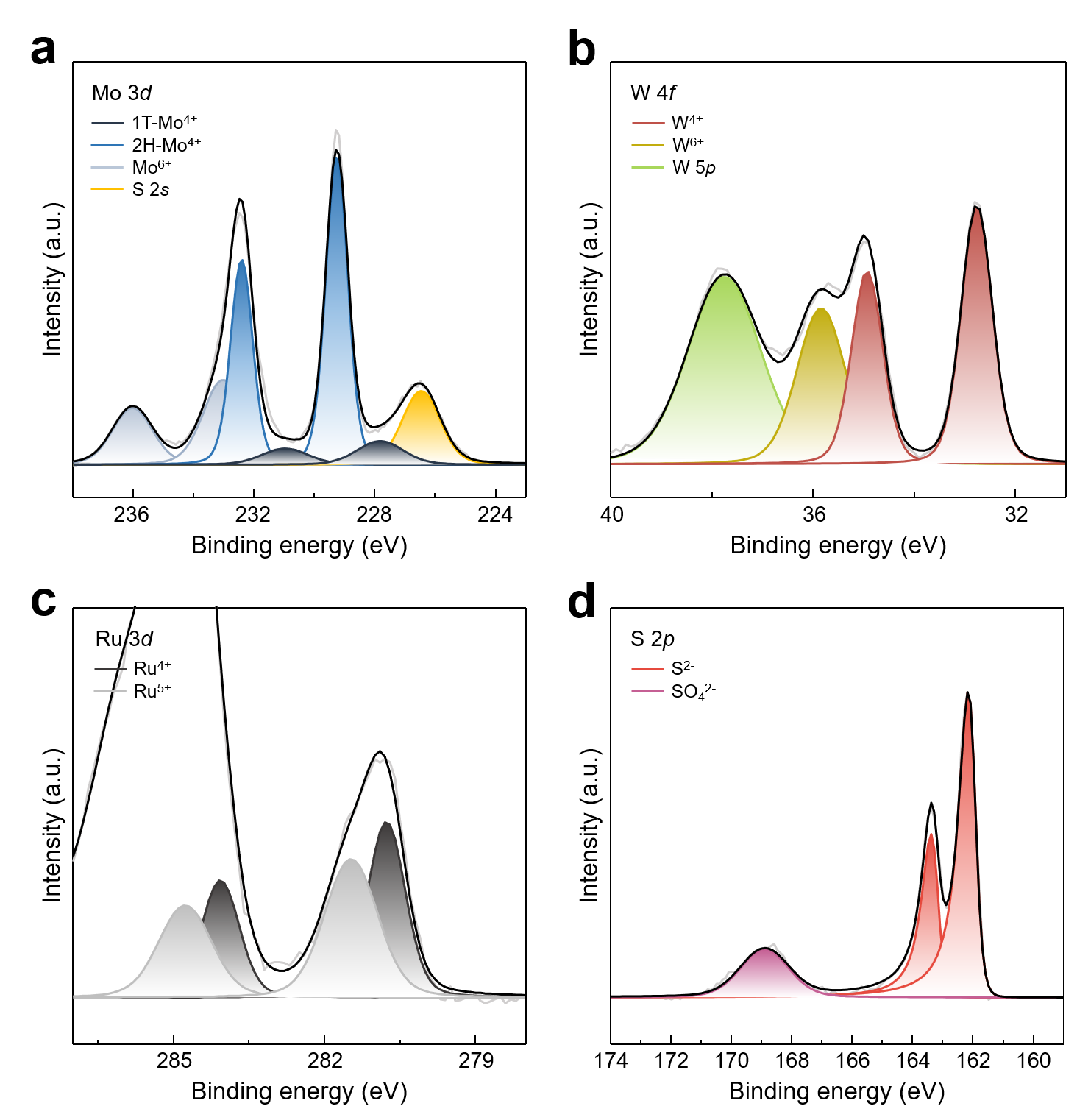


**Figure S14 |** (a) Mo 3*d*, (b) W 4*f*, (c) Ru 3*d*, and (d) S 2*p* XPS spectra of (MoWRu)S_2_.

It is revealed that (MoWRu)S_2_ possesses a lower proportion of 1T-Mo^4+^ than that in (MoWTaNbRu)S_2_.


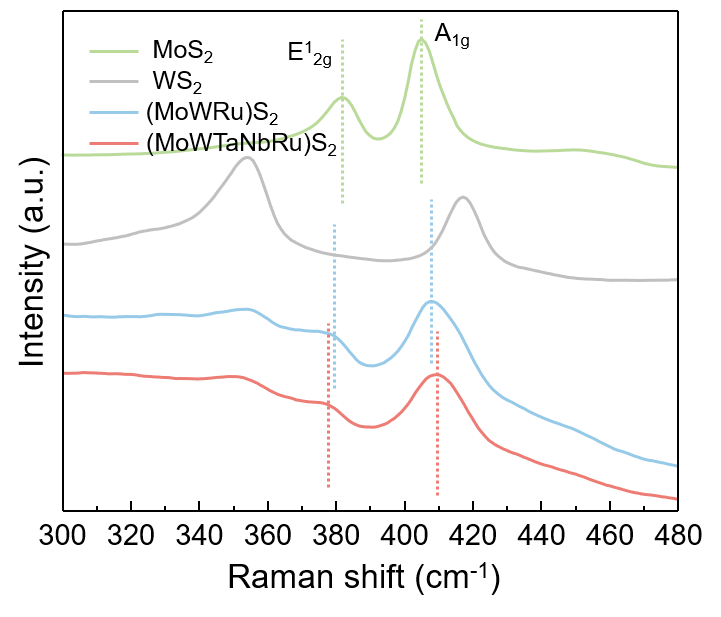


**Figure S15 |** Raman spectra of MoS_2_, WS_2_, (MoWRu)S_2_, and (MoWTaNbRu)S_2_.


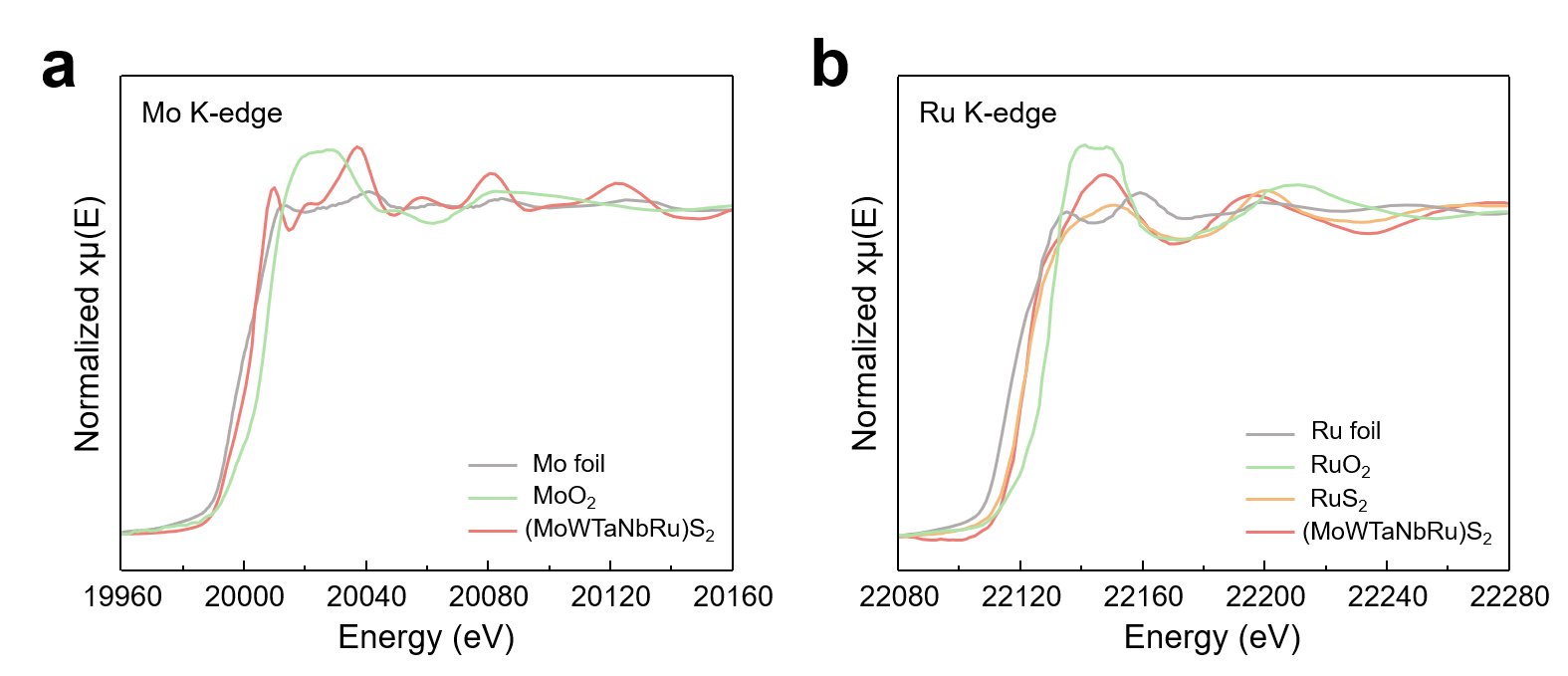


**Figure S16 |** (a) Mo K-edge and (b) Ru K-edge XANES spectra.

The energy absorption threshold of (MoWTaNbRu)S_2_ is positioned between that of metal foil and metal oxide, indicating oxidized state of Mo and Ru species in (MoWTaNbRu)S_2_. The near-edge absorption energy of (MoWTaNbRu)S_2_ at the Ru K-edge shifts slightly to higher energy compared to RuS_2_, suggesting that Ru serves as an electron donor.


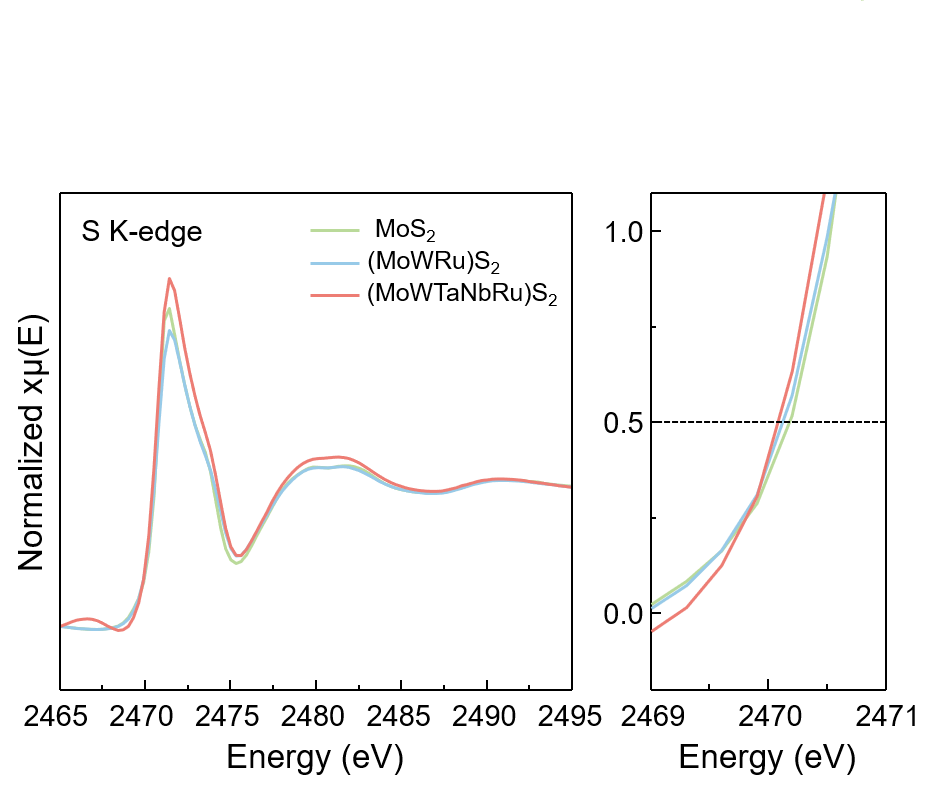


**Figure S17 |** S K-edge XANES spectra of MoS_2_, (MoWRu)S_2_, and (MoWTaNbRu)S_2_.


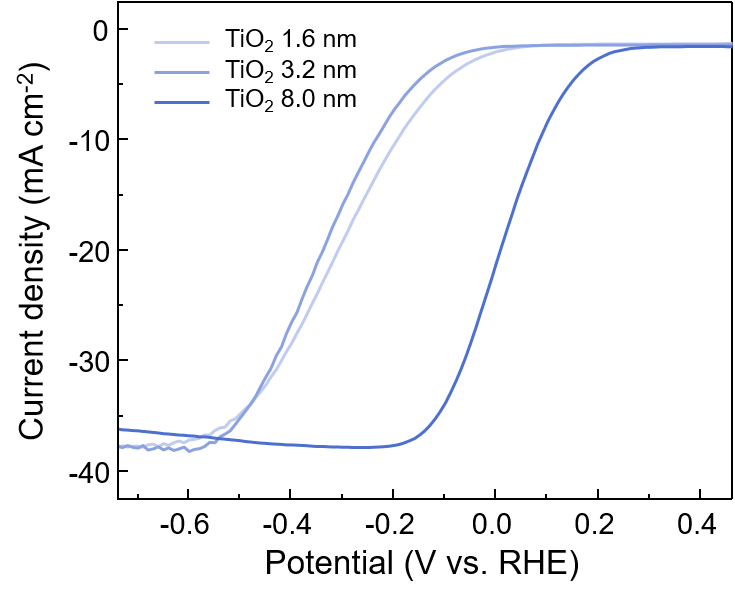


**Figure S18 |** LSV curves for PEC-HER with varying thicknesses of the TiO_2_ passivation layer.


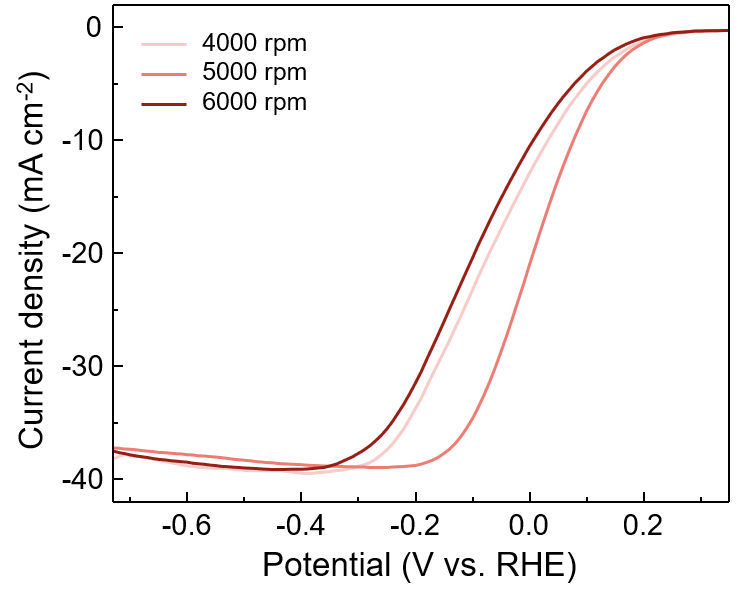


**Figure S19 |** LSV curves for PEC-HER with varying the rotating speed during spin-coating of precursor solution for high-entropy (MoWTaNbRu)S_2_ while maintaining the same composition and crystallization conditions.

For thicker film (4000 rpm), the increased thickness prolongs the photogenerated electron transport pathway from *p*-Si to the electrolyte, resulting in higher charge transport resistance. In contrast, for thinner film (6000 rpm), incomplete surface coverage of the underlying *p*-Si photoabsorber results in increased surface recombination and insufficient catalytic site density, both of which limit the overall photocurrent. These results demonstrate that the 5 nm (MoWTaNbRu)S_2_ thin film provides the best compromise between interfacial charge transfer and catalytic surface coverage, maximizing PEC-HER performance.


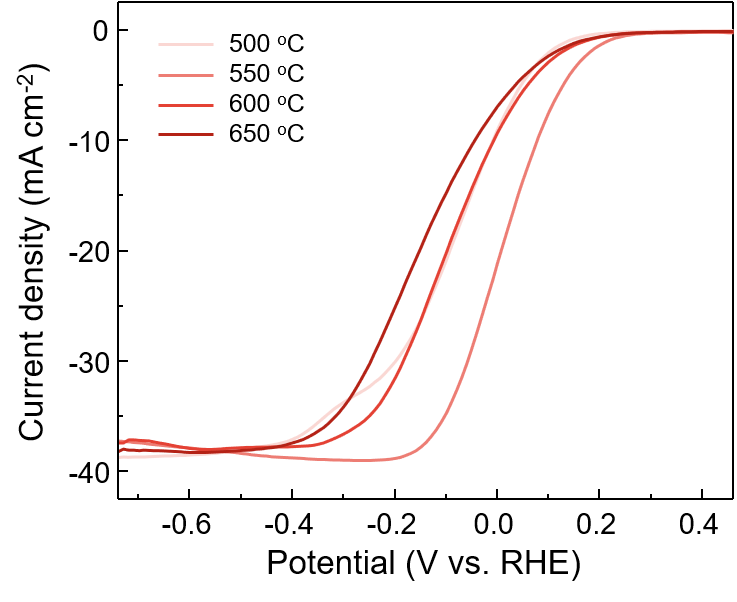


**Figure S20 |** LSV curves for PEC-HER with varying sulfurization temperatures.


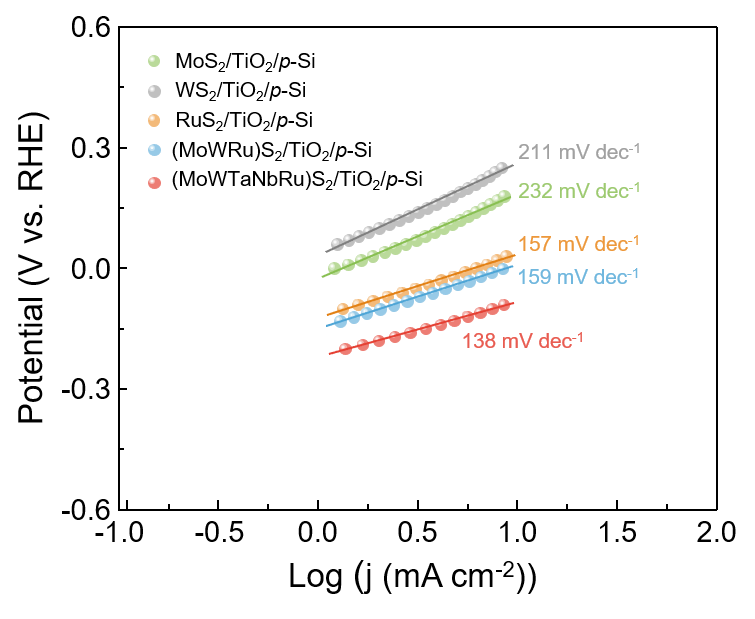


**Figure S21 |** Tafel plots of the photocathodes with MoS_2_, WS_2_, RuS_2_, (MoWRu)S_2_, and (MoWTaNbRu)S_2_.


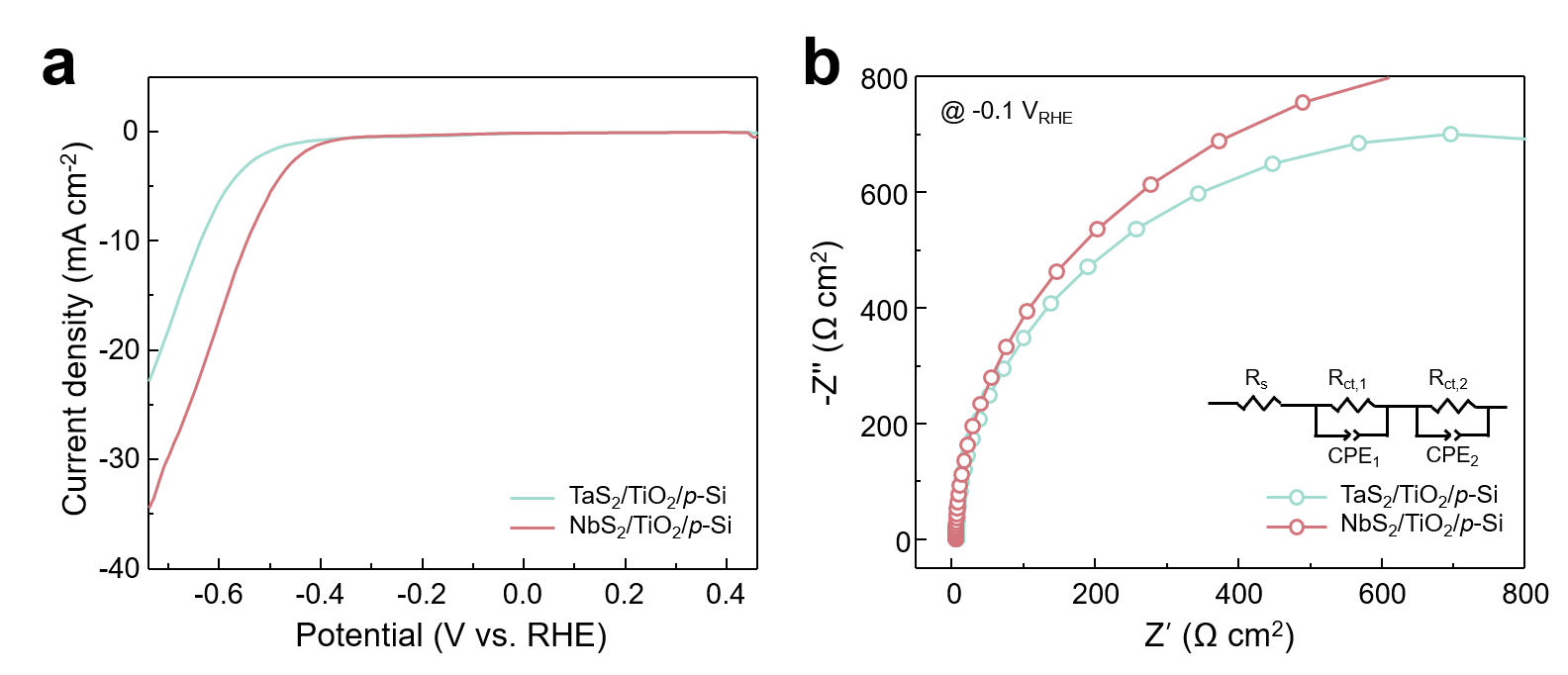


**Figure S22 |** (a) LSV and (b) PEIS plots of the photocathodes with TaS_2_ and NbS_2_ in 0.5 M H_2_SO_4_ electrolyte.


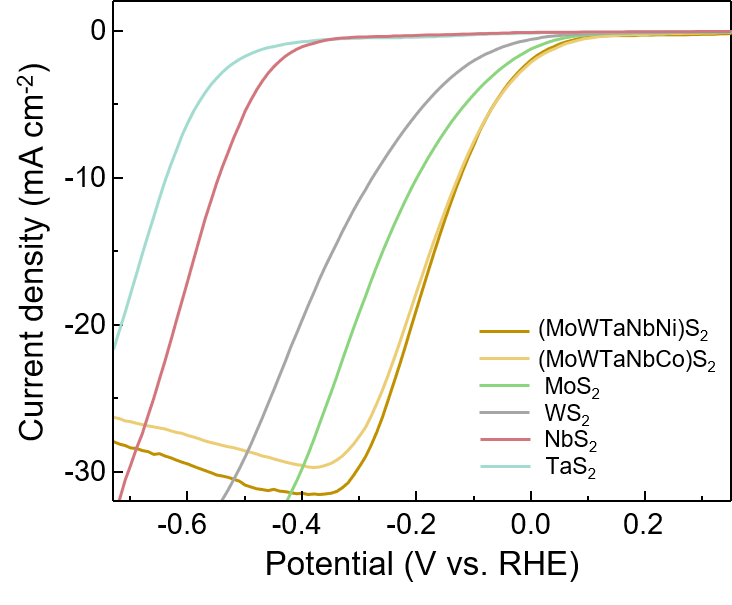


**Figure S23 |** LSV curves of photocathodes with (MoWTaNbNi)S_2_, (MoWTaNbCo)S_2_, and single-metal TMDs.


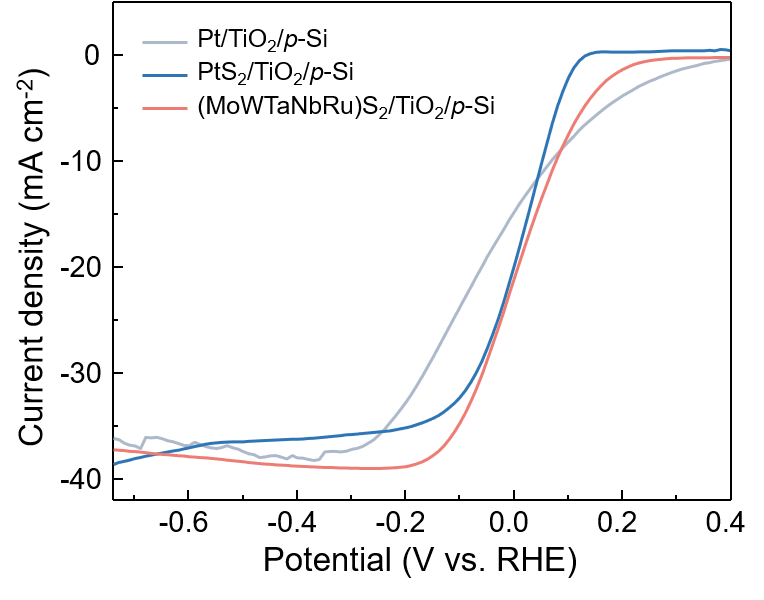


**Figure S24 |** LSV curves of photocathodes with Pt, PtS_2_, and (MoWTaNbRu)S_2_ thin-film catalysts.


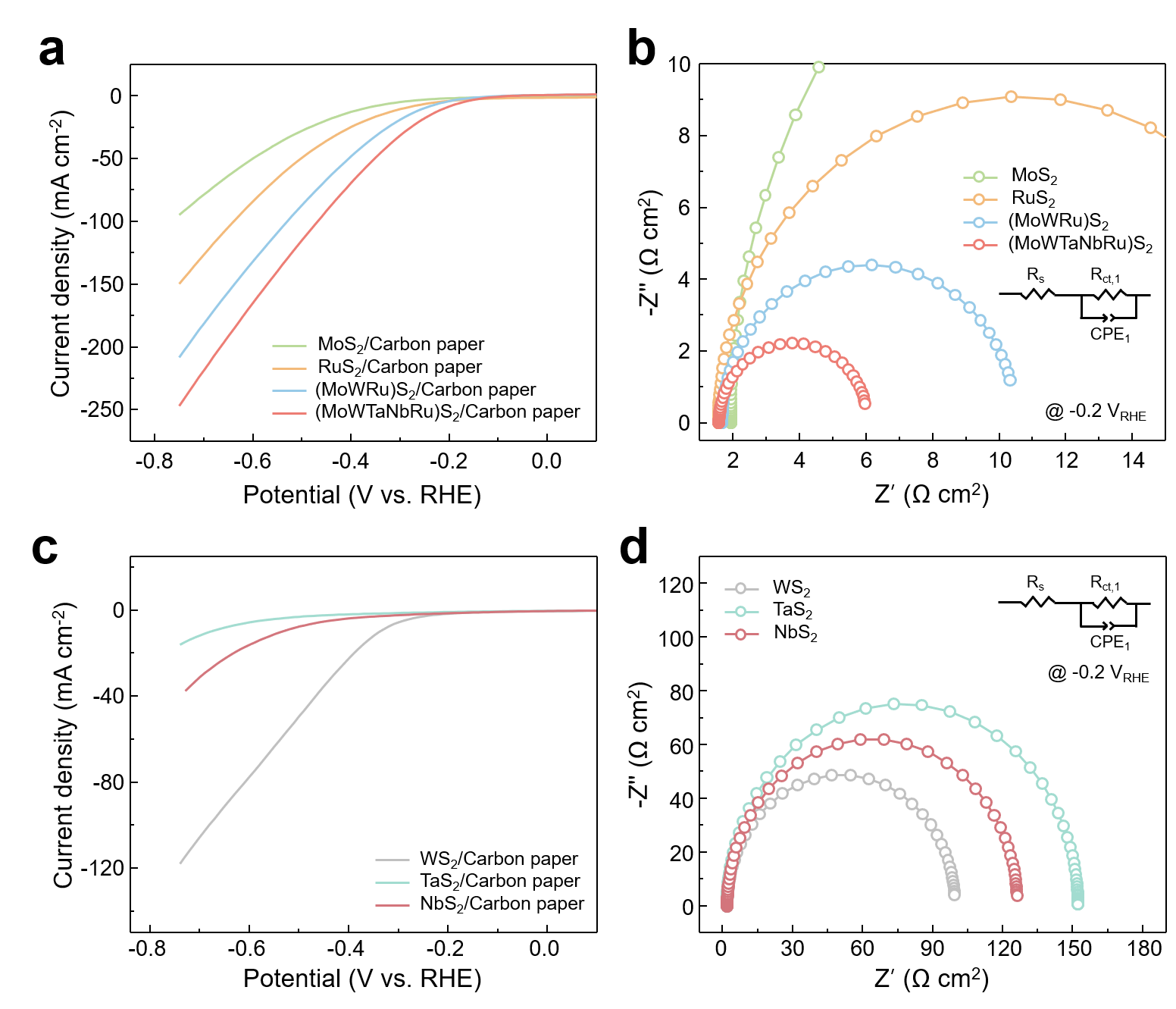


**Figure S25 |** Electrochemical measurements of TMDs deposited on carbon paper. (a) LSV curves and (b) EIS spectra of MoS_2_, RuS_2_, (MoWRu)S_2_, and (MoWTaNbRu)S_2_. (c) LSV curves and (d) EIS spectra of WS_2_, TaS_2_, and NbS_2_.

The (MoWTaNbRu)S_2_ achieves the highest current density at whole potential range and the lowest interfacial charge transfer resistance.

**
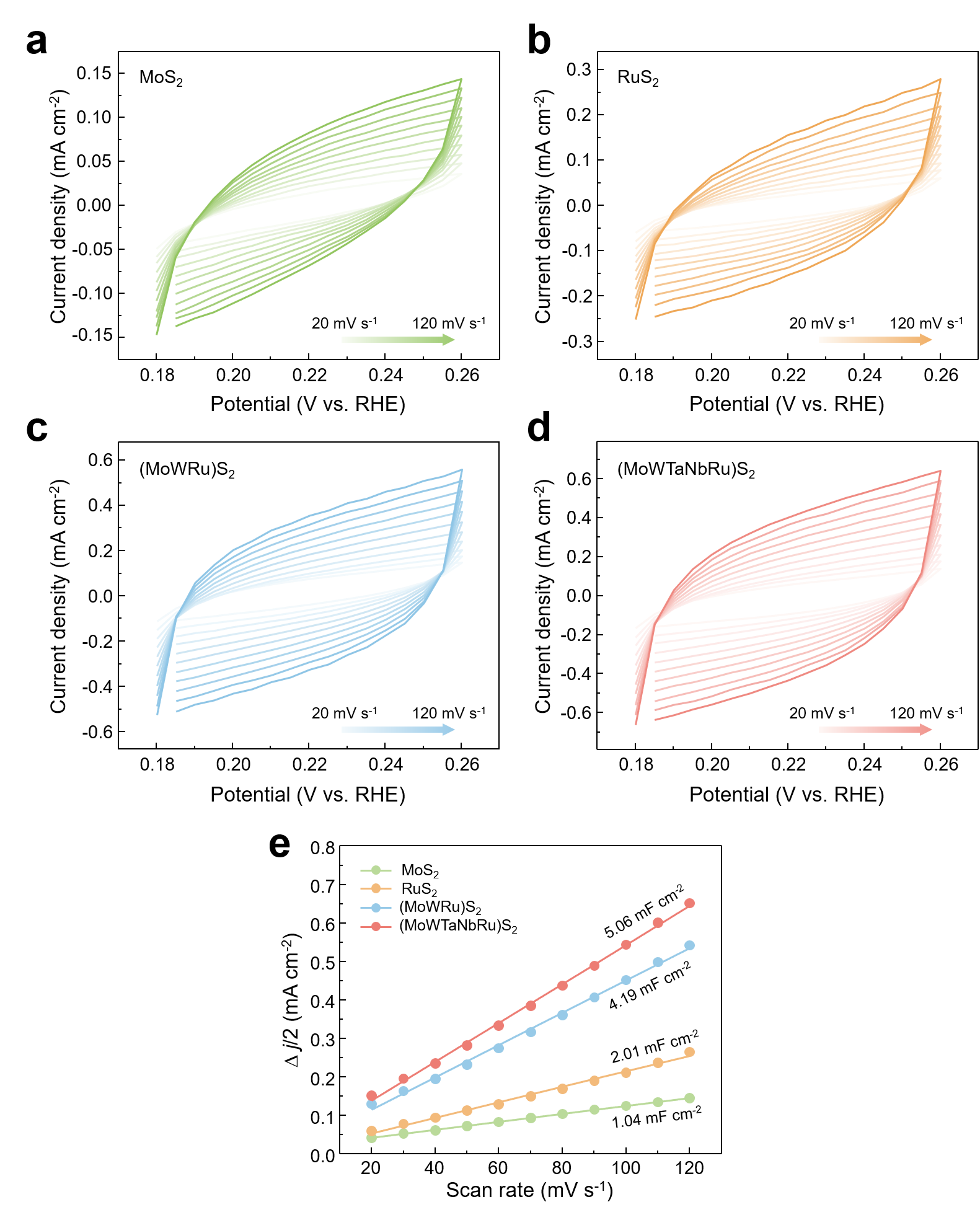
**

**Figure S26 |** Cyclic voltammogram (CV) curves recorded at different scan rates (20 to 120 mV s^-1^) of (a) MoS_2_/carbon paper, (b) RuS_2_/carbon paper, (c) (MoWRu)S_2_/carbon paper, and (d) (MoWTaNbRu)S_2_/carbon paper. (e) Electrochemical double-layer capacitances of MoS_2_, RuS_2_, (MoWRu)S_2_, and (MoWTaNbRu)S_2_.

The electrochemical double-layer capacitances (C_dl_) are calculated to reveal the electrochemically active surface area (ECSA). The (MoWTaNbRu)S_2_ shows the largest value of C_dl_ (5.06 mF cm^-2^) among the samples, implying that it possesses the largest amount of electrochemically active sites.


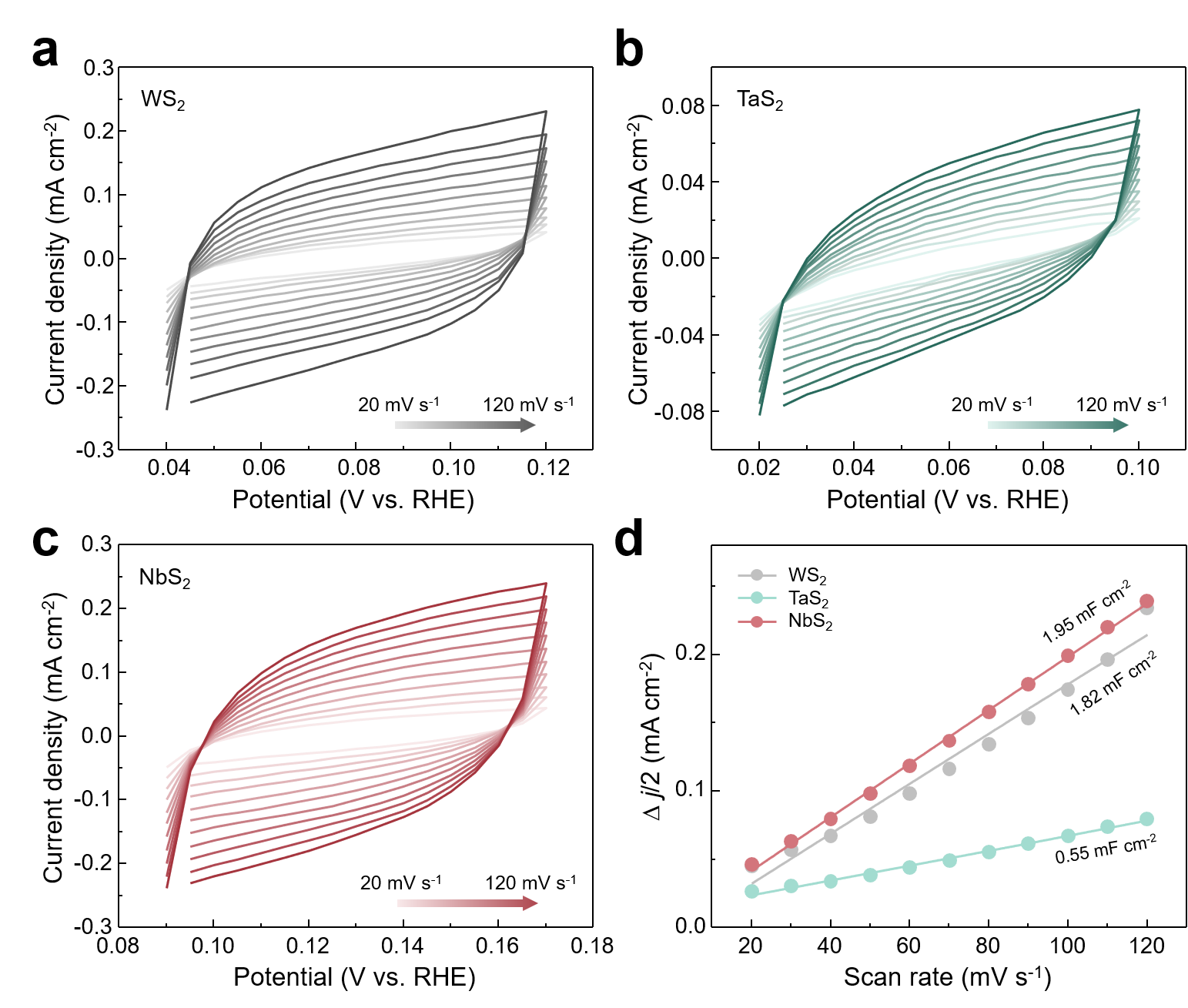


**Figure S27 |** Cyclic voltammogram (CV) curves recorded at different scan rates (20 to 120 mV s^-1^) of (a) WS_2_/carbon paper, (b) TaS_2_/carbon paper, and (c) NbS_2_/carbon paper. (d) Electrochemical double-layer capacitances of WS_2_, TaS_2_, and NbS_2_.


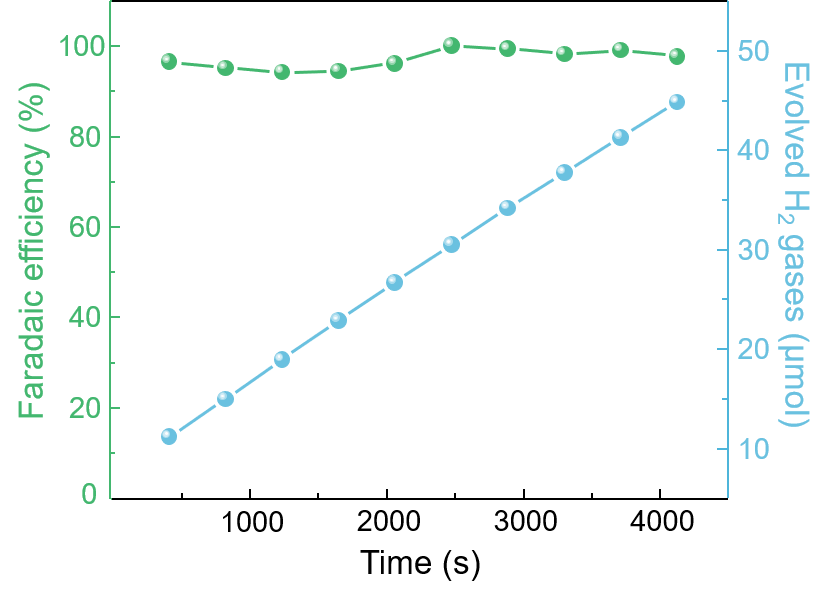


**Figure S28 |** Faradaic efficiency and evolved hydrogen gas of (MoWTaNbRu)S_2_ during PEC-HER.


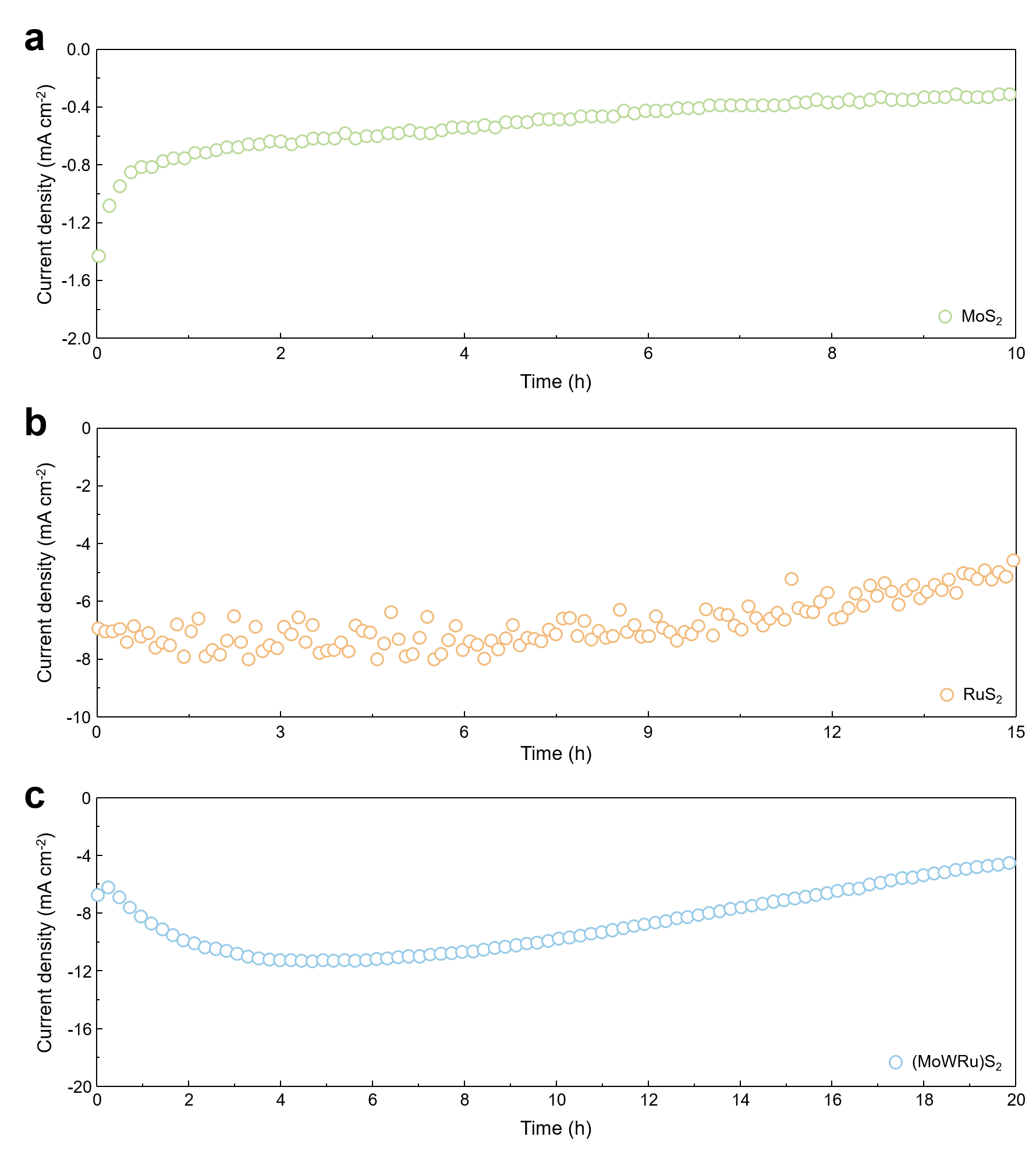


**Figure S29 |** Photoelectrochemical stability test of (a) MoS_2_, (b) RuS_2_, and (c) (MoWRu)S_2_ by chronoamperometry at the applied potential of 0 V versus RHE.


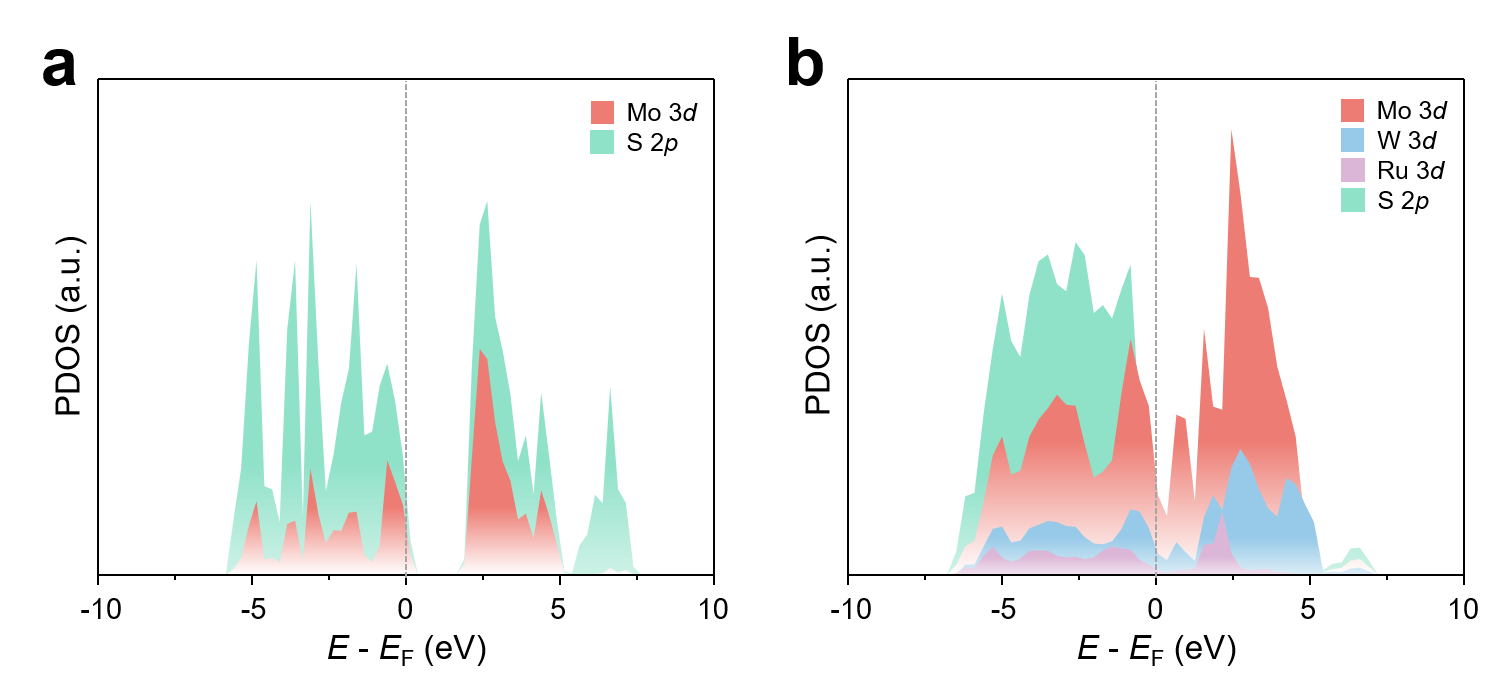


**Figure S30 |** Partial density of states (PDOSs) of (a) MoS_2_ and (b) (MoWRu)S_2_.


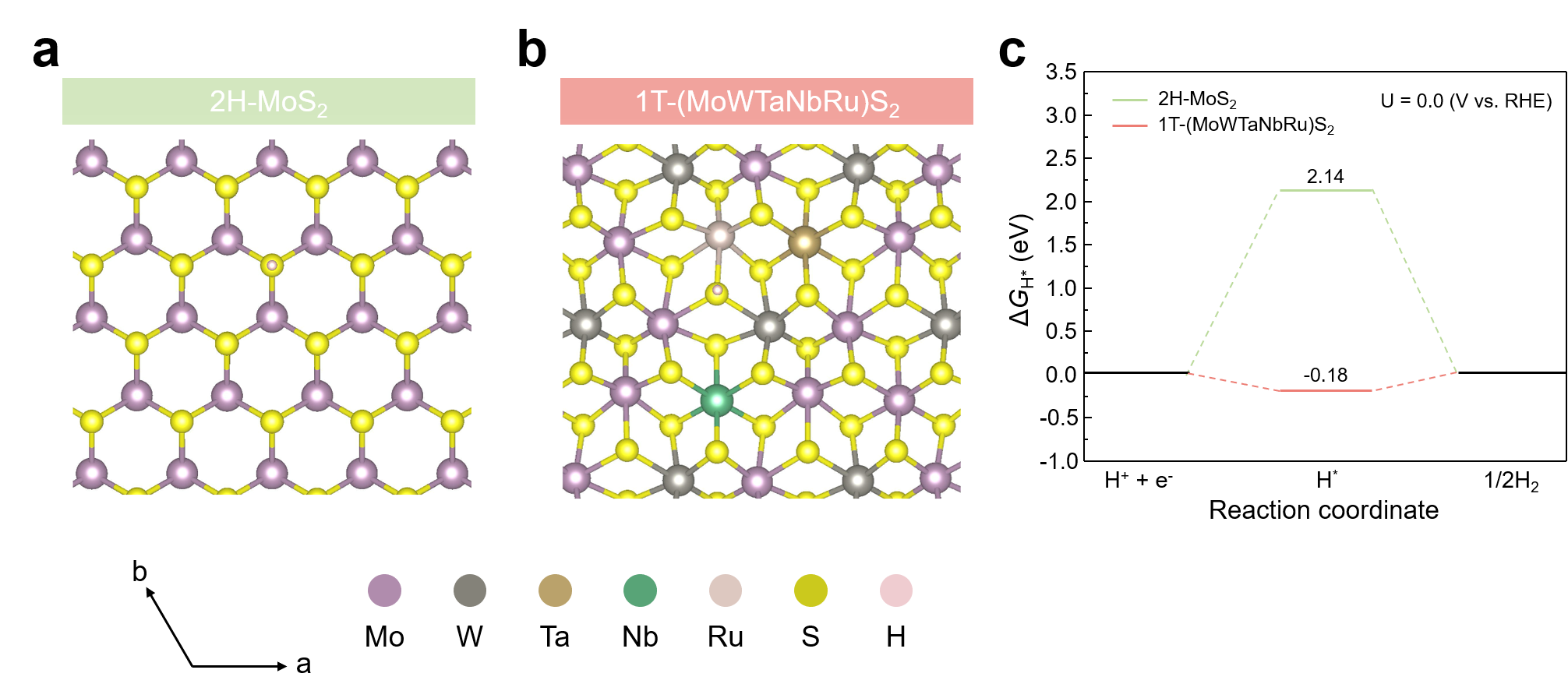


**Figure S31 |** Atomic models of (a) 2H-MoS_2_ and (b) 1T-(MoWTaNbRu)S_2_ with H adsorption. (c) Hydrogen adsorption Gibbs free energies (ΔG_H*_) of 2H-MoS_2_ and 1T-(MoWTaNbRu)S_2_ calculated for the top S sites at U = 0 V versus RHE.


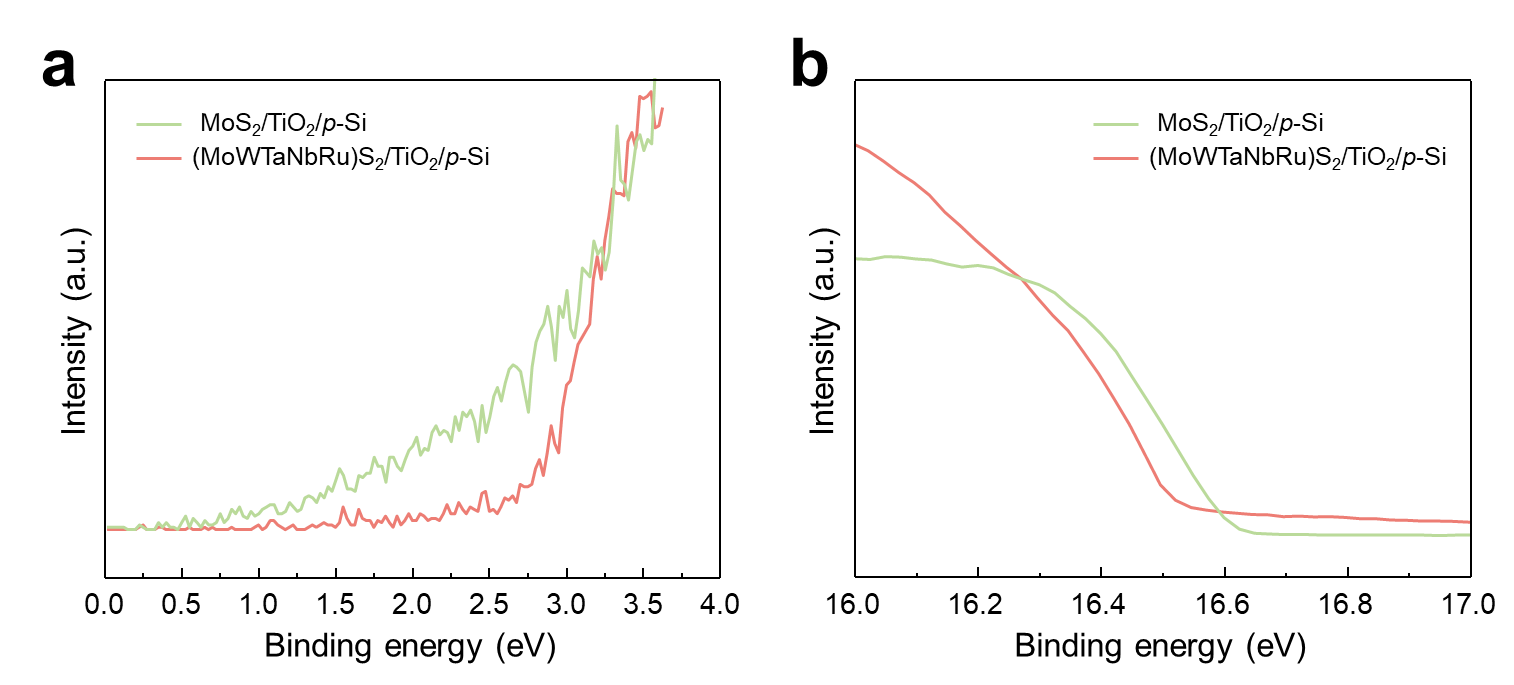


**Figure S32 |** (a) UPS and (b) XPS valence-band spectra as a function of binding energy for the MoS_2_/TiO_2_/*p*-Si and (MoWTaNbRu)S_2_/TiO_2_/*p*-Si.


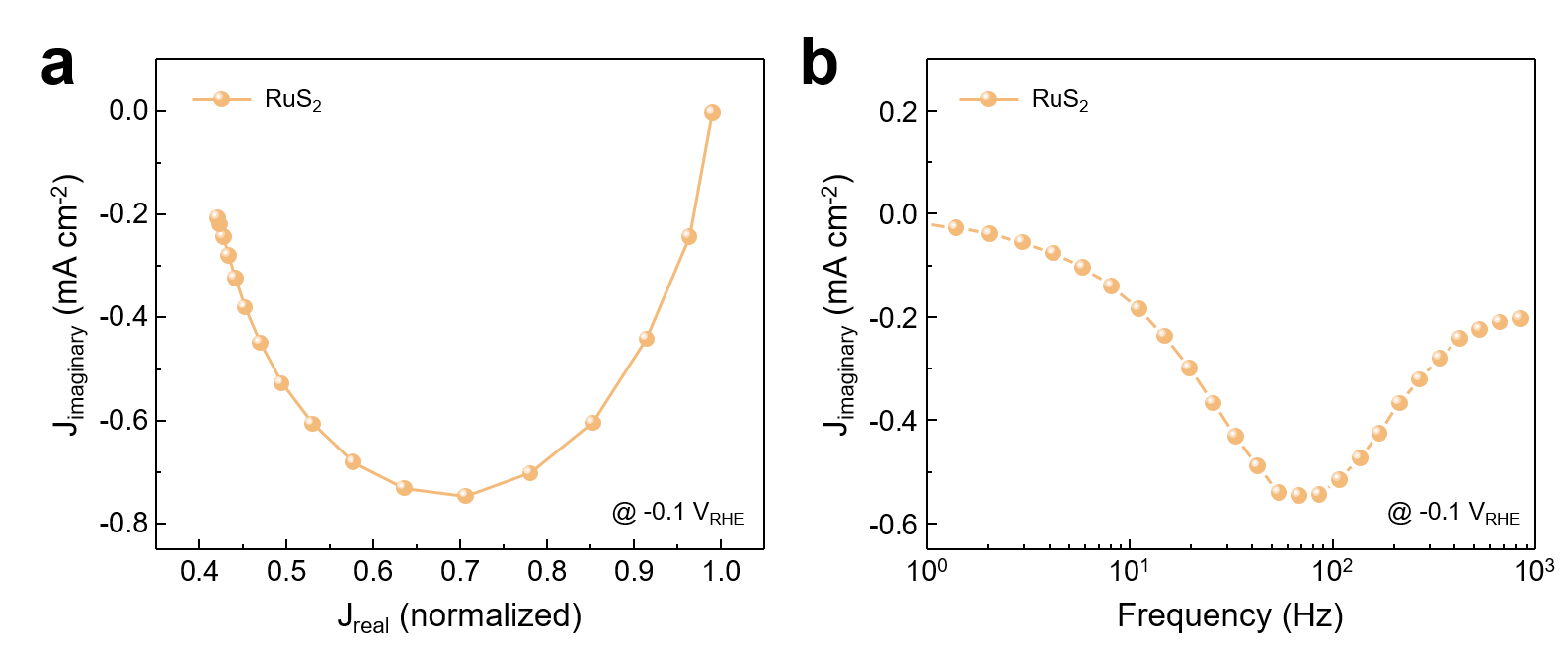


**Figure S33 |** (a) Intensity-modulated photocurrent spectroscopy (IMPS) Nyquist plot and (b) frequency dependent imaginary photocurrent plot of RuS_2_/TiO_2_/*p*-Si at the applied potential of -0.1 V versus RHE.


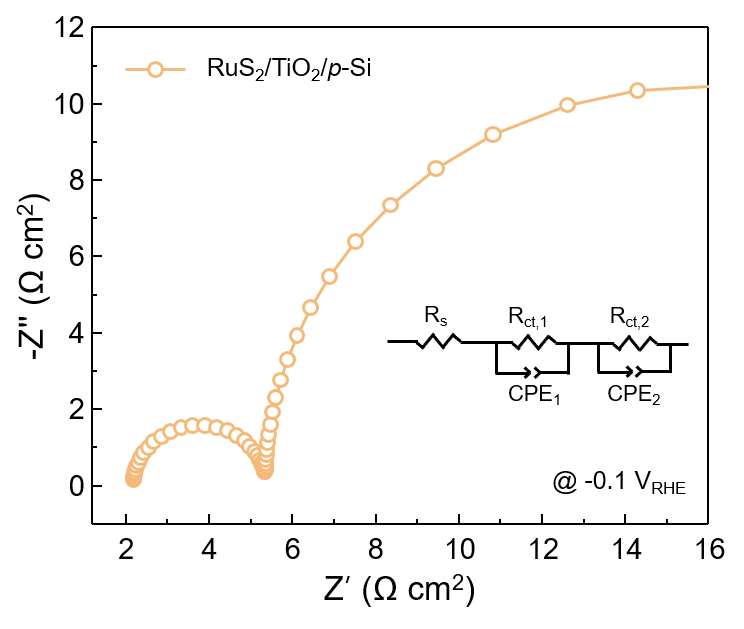


**Figure S34 |** Photoelectrochemical impedance spectroscopy plot of RuS_2_/TiO_2_/*p*-Si.


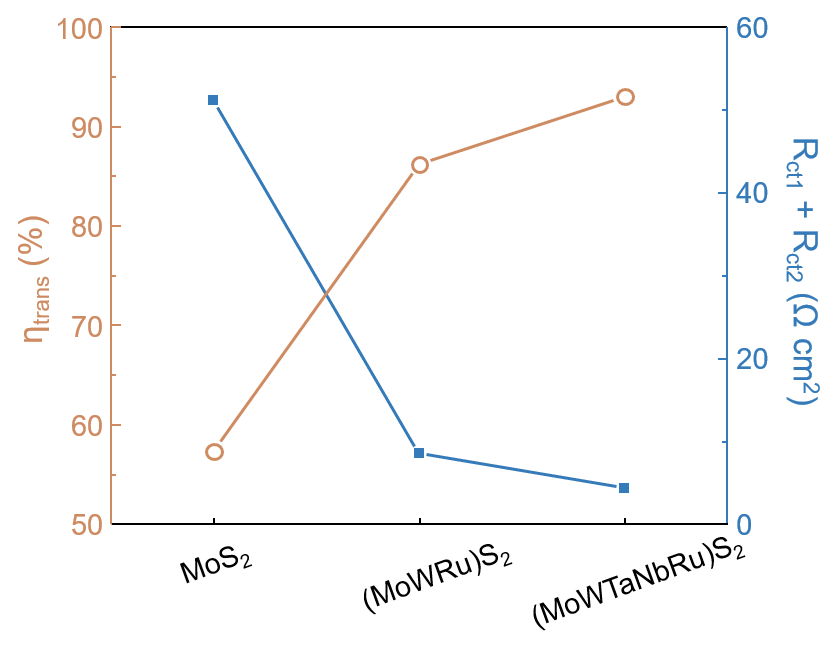


**Figure S35 |** Charge transfer efficiencies obtained from IMPS and charge transfer resistances at the interfaces obtained from PEIS of MoS_2_, (MoWRu)S_2_, and (MoWTaNbRu)S_2_.


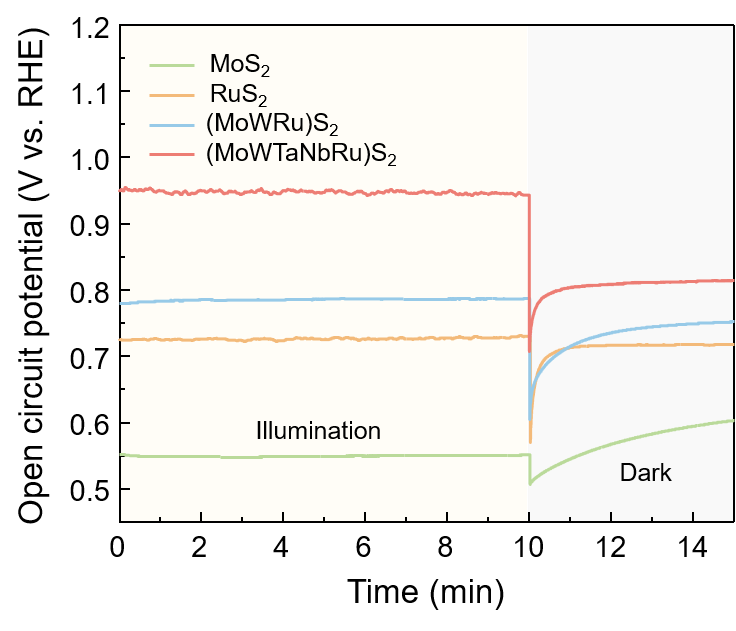


**Figure S36 |** Open circuit potential measurements in the dark condition and under illumination.


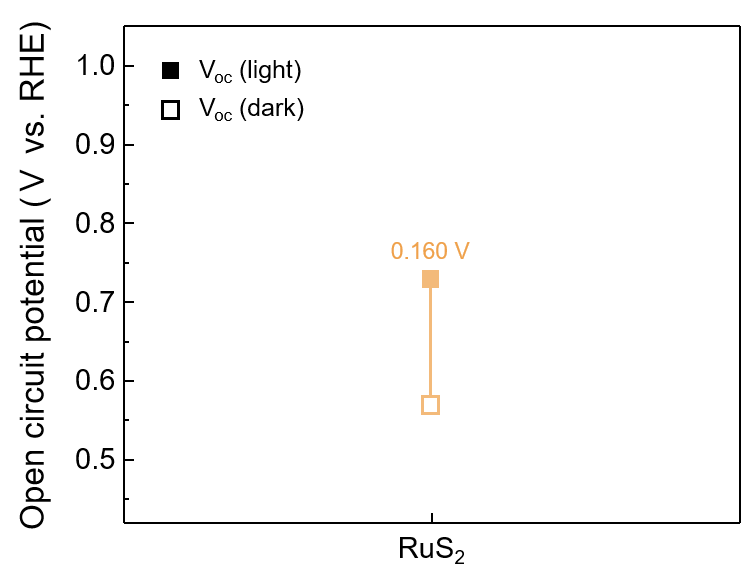


**Figure S37 |** Open circuit potential difference of RuS_2_/TiO_2_/*p*-Si.


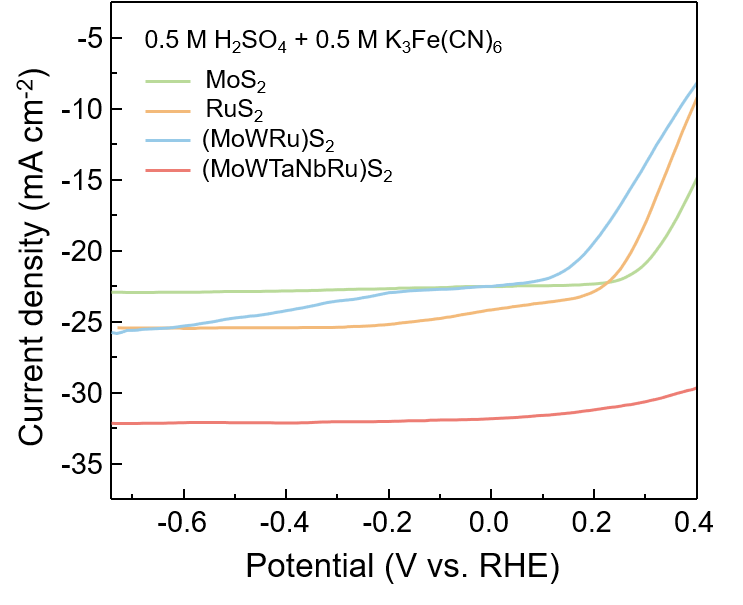


**Figure S38 |** J-V curves of photocathodes measured in 0.5 M H_2_SO_4_ + 0.5 M K_3_Fe(CN)_6_ electrolyte.

The ratio of the water reduction photocurrent density with and without the scavenger is charge injection efficiency.


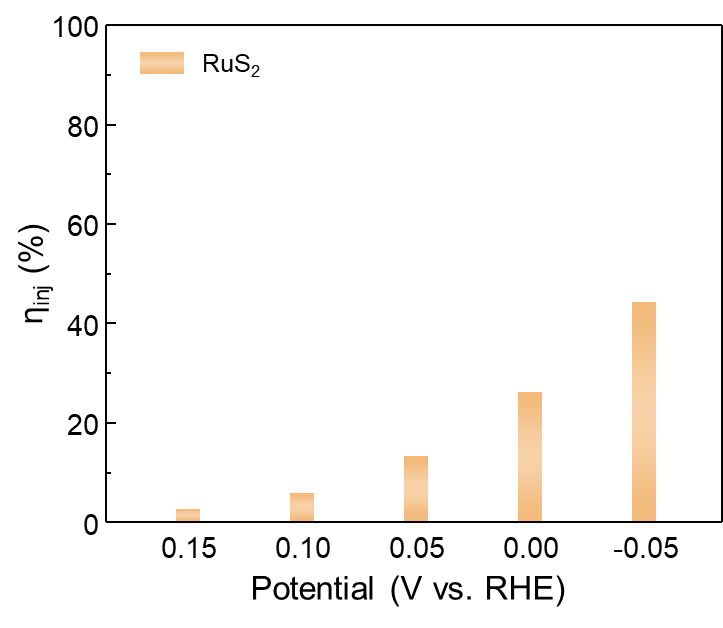


**Figure S39 |** Injection efficiency of RuS_2_/TiO_2_/*p*-Si.

**Supplementary Tables**

**Table S1 |** Synthesis of recently reported high-entropy transition metal chalcogenides.

| Material | Synthetic method | Scale | Temperature (^o^C) | Substrate | Reference |
| --- | --- | --- | --- | --- | --- |
| PbSbSnSeTeS | Ingot | N/A | 1150 | N/A | Science, 2021^3^ |
| NiFeMnInCoZnPSSe | Space-confined chemical vapour transport | ~ 50 μm | 1000 | SiO_2_/Si or Mica | Nat. Syn., 2025^4^ |
| (MoWNbTaV)S_2_ | High-temperature liquid-phase reaction | ~ 20 μm | 930 | Mica | J. Am. Chem. Soc., 2025^5^ |
| (WMoVScY)S_2_ | Conversion of *i*-MAX | ~ 10 nm | 1400 | N/A | Adv. Mater., 2025^6^ |
| (MoWVNbTa)S_2_ | Chemical vapor transport | ~ 600 nm | 1000 | N/A | Adv. Mater., 2021^7^ |
| (CrMnFeCoNi)S_x_ | Pulse thermal decomposition | 11.9 nm | 1377 | Carbon | Adv. Energy Mater., 2020^8^ |
| (VNbMoTaW)S_2_ | Ultrahigh vacuum sputtering | N/A | 788 ~ 1076 | Al_2_O_3_, hBN/Al_2_O_3_ | Nano Lett., 2024^9^ |
| (MoWVNbTa)S_2_ | NaCl-assisted chemical vapor deposition | ~ 10 μm | 900 | SiO_2_/Si | Chem. Eng. J., 2025^10^ |
| (FeCoCrNiMn)TiS_2_ | Chemical vapor transport | N/A | 1000 ~ 1100 | N/A | Acta Mater., 2021^11^ |
| **(MoWTaNbRu)S_2_** | **Thermolysis** | **4-inch wafer scale** | **550** | **SiO_2_/Si, Al_2_O_3_, TiO_2_/Si photoelectrode** | **Our work** |

**Table S2 |** Inductively coupled plasma-mass spectrometry (ICP-MS) measurement data.

| element | Mo | W | Ta | Nb | Ru |
| --- | --- | --- | --- | --- | --- |
| n (mmol/kg) | 0.0816 | 0.1235 | - | 0.0910 | 0.0632 |

Ta could not be reliably quantified by ICP-MS under our measurement conditions, which is attributed to its well-known resistance to complete dissolution during conventional acid digestion. In particular, Ta readily forms highly stable refractory species that are difficult to decompose, resulting in incomplete digestion and consequently low analytical recovery.

**Table S3 |** Contact angles and surface energies of photocathodes.

| Photocathode | Contact angle (^o^) | Surface energy (mN m^-1^) |
| --- | --- | --- |
| MoS_2_/TiO_2_/*p*-Si | 48.7 | 49.81 |
| RuS_2_/TiO_2_/*p*-Si | 51.9 | 48.03 |
| (MoWRu)S_2_/TiO_2_/*p*-Si | 23.8 | 61.93 |
| (MoWTaNbRu)S_2_/TiO_2_/*p*-Si | 21.4 | 62.84 |

**Table S4 |** Onset potentials and current densities at 0 V versus RHE of photocathodes.

| Photocathode | Onset potential  (V vs. RHE) | Current density at 0 V_RHE_  (mA cm^-2^) |
| --- | --- | --- |
| MoS_2_/TiO_2_/*p*-Si | 0.01 | -1.21 |
| RuS_2_/TiO_2_/*p*-Si | 0.12 | -6.26 |
| (MoWRu)S_2_/TiO_2_/*p*-Si | 0.15 | -8.46 |
| (MoWTaNbRu)S_2_/TiO_2_/*p*-Si | 0.22 | -21.10 |

**Table S5 |** Ru concentration measured by inductively coupled plasma-mass spectrometry (ICP-MS).

| Photocathode | Ru concentration (mg/kg) |
| --- | --- |
| RuS_2_/TiO_2_/*p*-Si | 1.66 |
| (MoWRu)S_2_/TiO_2_/*p*-Si | 0.38 |
| (MoWTaNbRu)S_2_/TiO_2_/*p*-Si | 0.68 |

| Photocathode | f at J_imag, max_ | η_trans_ | k_trans_ | k_rec_ |
| --- | --- | --- | --- | --- |
| MoS_2_/TiO_2_/*p*-Si | 63.1 | 57.3 | 396.47 | 227.18 |
| RuS_2_/TiO_2_/*p*-Si | 67.9 | 39.5 | 168.52 | 258.11 |
| (MoWRu)S_2_/TiO_2_/*p*-Si | 29.71 | 86.2 | 160.86 | 25.75 |
| (MoWTaNbRu)S_2_/TiO_2_/*p*-Si | 22.35 | 93.0 | 130.61 | 9.83 |

**Table S6 |** Frequency at the maximum imaginary current, charge transfer efficiency, charge transfer constant, and charge recombination constant.

**Table S7 |** Fitted interfacial charge transport resistances obtained by PEIS.

| Resistance  Photocathode | R_ct,1_  (Ω cm^2^)  [*p*-Si → surface] | R_ct,2_  (Ω cm^2^)  [surface → electrolyte] |
| --- | --- | --- |
| MoS_2_/TiO_2_/*p*-Si | 45.60 | 5.65 |
| RuS_2_/TiO_2_/*p*-Si | 22.00 | 5.05 |
| (MoWRu)S_2_/TiO_2_/*p*-Si | 6.31 | 2.37 |
| (MoWTaNbRu)S_2_/TiO_2_/*p*-Si | 2.68 | 1.82 |

**Supplementary Methods**

**DFT calculations**

All the spin-polarized density functional theory (DFT) calculations were conducted using the Vienna Ab-initio Simulation Package (VASP) with the projector augmented wave method for the core region and a plane-wave kinetic energy cutoff of 450 eV^12–14^. The generalized gradient approximation (GGA) in the form of Perdew-Burke-Ernzerhof (PBE) for the exchange-correlation potentials was used^15,16^. Dispersion interactions were included using DFT-D3 dispersion correction method^17^. The large vacuum layers of these slab models were set at least 15 Å in the z direction to isolate the surface and prevent interaction between two periodic units. A 3×3×1 Gamma-centered Monkhorst-pack sampled k-point grid was employed to sample the reciprocal space for the slab models. The atomic layers and adsorbates are free to move in all directions until the convergence of energy and residual atomic force on each atom was less than 1×10^-4^ eV and 0.02 eV Å^-1^, respectively. Calculations on HER free energy diagram were conducted at the surface of monolayer structures. The free energy of the hydrogen adsorption is calculated by ΔG_H_ = ΔE_H_ + ΔE_ZPE_ – TΔS_H_ where ΔE_H_ is the hydrogen chemisorption energy defined by ΔE_H_ = E_sur_ − H − E_sur_ + 1/2E_H2_, and ΔE_ZPE_ is the difference of the zero point energy between the adsorbed state and the gas phase.

**Calculations of k_trans_ and k_rec_.**

<RuS_2_/TiO_2_/*p*-Si @ -0.1 V_RHE_>

k_trans_ + k_rec_ = 2πf = 2π(67.9) = 426.63

k_trans_ = transfer efficiency × (k_trans_ + k_rec_) = 0.395 × 426.63 = 168.52

k_rec_ = 426.63 - 168.52 = 258.11

<(MoWRu)S_2_/TiO_2_/*p*-Si @ -0.1 V_RHE_>

k_trans_ + k_rec_ = 2πf = 2π(29.71) = 186.61

k_trans_ = transfer efficiency × (k_trans_ + k_rec_) = 0.862 × 186.61 = 160.86

k_rec_ = 186.61 – 160.86 = 25.75

<(MoWTaNbRu)S_2_/TiO_2_/*p*-Si @ -0.1 V_RHE_>

k_trans_ + k_rec_ = 2πf = 2π(22.35) = 140.74

k_trans_ = transfer efficiency × (k_trans_ + k_rec_) = 0.93 × 140.74 = 130.89

k_rec_ = 140.74 – 130.89 = 9.85

**Supplementary References**

1. Shi, J. *et al.* Two-dimensional metallic tantalum disulfide as a hydrogen evolution catalyst. *Nat. Commun.* **8**, 958 (2017).

2. Peng, Y. *et al.* Highly Stable Vertically Oriented 2H-NbS2 Nanosheets on Carbon Nanotube Films toward Superior Electrocatalytic Activity. *Adv. Energy Mater.* **14**, 2302510 (2024).

3. Jiang, B. *et al.* High-entropy-stabilized chalcogenides with high thermoelectric performance. *Science* **371**, 830–834 (2021).

4. Que, H. *et al.* Synthesis of two-dimensional transition metal phosphorous chalcogenides and their high-entropy alloys. *Nat. Synth.* **4**, 582–591 (2025).

5. Wang, Z. *et al.* Synthesis of Two-Dimensional High-Entropy Transition Metal Dichalcogenide Single Crystals. *J. Am. Chem. Soc.* **147**, 1392–1398 (2025).

6. Wang, H. *et al.* High-Entropy 1T-Phase Quantum Sheets of Transition-Metal Disulfides. *Adv. Mater.* **37**, 2500321 (2025).

7. Cavin, J. *et al.* 2D High-Entropy Transition Metal Dichalcogenides for Carbon Dioxide Electrocatalysis. *Adv. Mater.* **33**, 2100347 (2021).

8. Cui, M. *et al.* High‐Entropy Metal Sulfide Nanoparticles Promise High‐Performance Oxygen Evolution Reaction. *Adv. Energy Mater.* **11**, 2002887 (2021).

9. Tanaka, K. *et al.* Growth of Highly Oriented (VNbMoTaW)S2 Layers. *Nano Lett.* **24**, 493–500 (2024).

10. Chin, C.-W. *et al.* Entropy-stabilized multi-element 2D TMDs: Tunable electronics and enhanced thermal stability. *Chem. Eng. J.* **524**, 169215 (2025).

11. Chen, H. *et al.* High-entropy structure design in layered transition metal dichalcogenides. *Acta Mater.* **222**, 117438 (2022).

12. Kresse, G. & Hafner, J. *Ab initio* molecular dynamics for liquid metals. *Phys. Rev. B* **47**, 558–561 (1993).

13. Kresse, G. & Furthmüller, J. Efficient iterative schemes for *ab initio* total-energy calculations using a plane-wave basis set. *Phys. Rev. B* **54**, 11169–11186 (1996).

14. Kresse, G. & Furthmüller, J. Efficiency of ab-initio total energy calculations for metals and semiconductors using a plane-wave basis set. *Comput. Mater. Sci.* **6**, 15–50 (1996).

15. Blöchl, P. E. Projector augmented-wave method. *Phys. Rev. B* **50**, 17953–17979 (1994).

16. Perdew, J. P., Burke, K. & Ernzerhof, M. Generalized Gradient Approximation Made Simple. *Phys. Rev. Lett.* **77**, 3865–3868 (1996).

17. Grimme, S., Antony, J., Ehrlich, S. & Krieg, H. A consistent and accurate ab initio parametrization of density functional dispersion correction (DFT-D) for the 94 elements H-Pu. *J. Chem. Phys.* **132**, 154104 (2010).
